# Revision of the Australian species of the weevil genus *Trigonopterus* Fauvel

**DOI:** 10.3897/zookeys.556.6126

**Published:** 2016-01-21

**Authors:** Alexander Riedel, Rene Tänzler

**Affiliations:** 1 State Museum of Natural History Karlsruhe, Erbprinzenstr. 13, D-76133 Karlsruhe, Germany; 2 Zoological State Collection, Münchhausenstr. 21, D-81247 Munich, Germany

**Keywords:** Australian Wet Tropics, Coleoptera, conservation, *cox1*, Curculionidae, Cryptorhynchinae, DNA barcoding, endemism, hyperdiverse, integrative taxonomy, morphology, weevils

## Abstract

The Australian species of the genus *Trigonopterus* Fauvel are revised. Eight previously recognized species are redescribed and 24 additional new species are described: *Trigonopterus allaetus* Riedel, **sp. n.**, *Trigonopterus athertonensis* Riedel, **sp. n.**, *Trigonopterus australinasutus* Riedel, **sp. n.**, *Trigonopterus australis* Riedel, **sp. n.**, *Trigonopterus bisignatus* Riedel, **sp. n.**, *Trigonopterus bisinuatus* Riedel, **sp. n.**, *Trigonopterus boolbunensis* Riedel, **sp. n.**, *Trigonopterus cooktownensis* Riedel, **sp. n.**, *Trigonopterus daintreensis* Riedel, **sp. n.**, *Trigonopterus deplanatus* Riedel, **sp. n.**, *Trigonopterus finniganensis* Riedel, **sp. n.**, *Trigonopterus fraterculus* Riedel, **sp. n.**, *Trigonopterus garradungensis* Riedel, **sp. n.**, *Trigonopterus hasenpuschi* Riedel, **sp. n.**, *Trigonopterus hartleyensis* Riedel, **sp. n.**, *Trigonopterus kurandensis* Riedel, **sp. n.**, *Trigonopterus lewisensis* Riedel, **sp. n.**, *Trigonopterus montanus* Riedel, **sp. n.**, *Trigonopterus monteithi* Riedel, **sp. n.**, *Trigonopterus mossmanensis* Riedel, **sp. n.**, *Trigonopterus oberprieleri* Riedel, **sp. n.**, *Trigonopterus robertsi* Riedel, **sp. n.**, *Trigonopterus terraereginae* Riedel, **sp. n.**, *Trigonopterus yorkensis* Riedel, **sp. n.**. All new species are authored by the taxonomist-in-charge, Alexander Riedel. Lectotypes are designated for the following names: *Idotasia aequalis* Pascoe, *Idotasia albidosparsa* Lea, *Idotasia evanida* Pascoe, *Idotasia laeta* Lea, *Idotasia rostralis* Lea, *Idotasia sculptirostris* Lea, *Idotasia squamosa* Lea. A new combination of the name *Idotasia striatipennis* Lea is proposed: *Trigonopterus striatipennis* (Lea), **comb. n.**. A key to the species is provided. Australian *Trigonopterus* occur in coastal Queensland, narrowly crossing into New South Wales. The southern parts of the range are inhabited by species found on foliage. A rich fauna of 19 edaphic species inhabiting the leaf litter of tropical forests is reported for the first time from the Australian Wet Tropics.

## Introduction

*Trigonopterus* Fauvel is a genus of wingless weevils of the subfamily Cryptorhynchinae (Alonso-Zarazaga and Lyal 1999) and highly species-rich in the tropical forests of southeast Asia and Melanesia (Tänzler et al. 2012). New Guinea appears to be the center of its diversity, with more than 300 species recorded (Riedel 2010, Tänzler et al. 2012). *Trigonopterus* is currently the subject of studies on its ecology (Riedel et al. 2010, Tänzler et al. 2012), biogeography (Tänzler et al. 2014, Tänzler et al. 2016) and functional morphology (van de Kamp et al. 2011, van de Kamp et al. 2014, van de Kamp et al. 2015). The need for a stable taxonomy with valid names became urgent, and a fast-lane approach of taxonomy (Riedel et al. 2013a) was established to describe 200 new species from Indonesia and Papua New Guinea (Riedel et al. 2013b, 2014).

The number of Australian *Trigonopterus* species is relatively small. Nevertheless, from a biogeographical perspective this continental and presumably old fauna is of great interest. Eight species have been described from Queensland to date (Pullen et al. 2014). A study of museum collections and a limited amount of field work resulted in the discovery of a modest number of additional, undescribed species. Many of them were first collected by Geoff Monteith (QMBA), who also discovered an edaphic group of species by sifting leaf litter. All the previously known Australian species had been collected from vegetation.

Despite these advances there are problems remaining. Some Australian *Trigonopterus* are difficult to characterize using morphological characters alone: species of the *Trigonopterus politus*-group (e.g., comprising the Australian *Trigonopterus aequalis* Pascoe, *Trigonopterus evanidus* Pascoe and *Trigonopterus albidosparsus* (Lea)) and of the *Trigonopterus squamosus*-group offer only few morphological characters, whereas molecular data indicate highly divergent lineages. Therefore we have to leave many museum specimens unidentified. Still, we believe that it is timely to present a first summary now, with the aims of 1) redescribing the known species based on their type material and 2) providing names for those new species that can be safely recognized based on morphological characters alone. Hopefully this study will instigate the additional field work needed to arrive at a more comprehensive understanding of the Australian *Trigonopterus* fauna.

## Materials and methods

This study is based on 673 specimens, including 11 type specimens of old collections. Some of the material was collected specifically for this project from vegetation with the help of a beating sheet, or by sifting the litter of primary forests and subsequent extraction of specimens from it using Winkler eclectors (Besuchet et al. 1987). DNA sequences were obtained for 86 of the freshly collected specimens. Holotypes of new species were selected from these sequence vouchers whenever possible. DNA was extracted nondestructively as described by Riedel et al. (2010). Type and other old specimens from collections were treated in the same way, which has also proved to be the most conservative method for the extraction of genitalia and at the same time allows saving the presumably more or less degraded DNA. Unfortunately all our trials of PCR using this old collection material failed, but it may be feasible in future with improved sequencing methods. The genitalia of most specimens did not require maceration after DNA extraction and could be directly stained in an alcoholic solution of chlorazol black and stored in glycerol in microvials attached to the pin of the specimens. Genitalia of specimens whose abdominal muscle tissue was not sufficiently digested after DNA extraction were macerated in a 10% KOH solution and rinsed in 3% acetic acid before staining. Illustrations of habitus and genitalia were prepared from holotypes. Finally, type series were supplemented with specimens stored in ethanol and older material from museum collections. Type depositories are cited using the following codens:

ANIC
Australian National Insect Collection, Canberra, Australia 


BMNH
 The Natural History Museum, London, UK 


QMBA
Queensland Museum, Brisbane, Australia 


SAMA
 South Australian Museum, Adelaide, Australia 


SMNK
Staatliches Museum für Naturkunde, Karlsruhe, Germany 


The methods applied for DNA sequencing and sequence analysis are described by Riedel et al. (2010) and Tänzler et al. (2012). Morphological descriptions are limited to major diagnostic characters, as outlined by Riedel et al. (2013a, b). Negative character states (i. e. the absence of a character) are only mentioned explicitly where this appears appropriate. For example, some species of the *Trigonopterus politus*-group have a weakly carinate dorsal margin of the eyes. In these cases the character is described, but for the majority of species, in which the eyes are dorsally simple and evenly rounded with the forehead, this condition is not mentioned. Common practice would require stating explicitly “eyes dorsally simple, rounded with forehead”. Although formally accurate, in groups comprising hundreds of species this leads to inflated descriptions that distract the reader from the important information by enumerating the absence of rare character states.

Morphological terminology follows Beutel and Leschen (2005) and Leschen et al. (2009), i.e. the terms “mesoventrite” / “metaventrite” are used instead of “mesosternum” / “metasternum”, and “mesanepisternum” / “metanepisternum” instead of “mesepisternum” / “metepisternum”; “penis” is used instead of “aedeagus” as the tegmen is usually without useful characters in *Trigonopterus* and therefore omitted from species descriptions. Descriptions were prepared using a Leica MZ16 dissecting microscope and a fluorescent-light desk lamp for illumination. Measurements were taken with the help of an ocular grid. The length of the body was measured in dorsal aspect from the elytral apex to the front of the pronotum. The width of the elytra was measured between the humeri at their greatest extent and across *both* elytra. Legs were described in an idealized laterally extended position; there is a dorsal / ventral and an anterior / posterior surface. Habitus illustrations were compiled using a DFC450 camera with L.A.S. 4.6.0 software adapted to a Z6 APO (all from Leica Microsystems, Heerbrugg, Switzerland). Photographic illustrations of genitalia were made using a JVC KY70 camera (JVC Professional Products) adapted to an Axio Imager M2 microscope (Carl Zeiss Microscopy), with 5× or 10× A-Plan lenses; the resulting image stacks were combined using the Helicon Focus 6.2.2 software (Helicon Soft Ltd). For photography the genitalia were temporarily embedded in glycerol gelatin, as described by Riedel (2005), with their longitudinal axis somewhat lifted anteriorly to adequately illustrate structures of the down-curved apex. All photographs were enhanced using Adobe Photoshop CS2 and CS6, but care was taken not to obscure or alter any features of the specimens illustrated.

Sequence data were submitted to the European Molecular Biology Laboratory (EMBL), and the accession numbers are provided under each species e.g. as “(EMBL # LN888232)”. Data on genetic material contained in this paper is published for non-commercial use only. Utilization for purposes other than non-commercial scientific research may infringe the conditions under which the genetic resources were originally accessed, and should not be undertaken without contacting the corresponding author of the paper and/or seeking permission from the original provider of the genetic material.

## Taxonomy

### Trigonopterus

Taxon classificationAnimaliaColeopteraCurculionidae

Fauvel, 1862

#### Type species.

*Trigonopterus insignis* Fauvel, 1862, by monotypy.

#### Diagnosis.

Fully apterous genus of Cryptorhynchinae. Length 1.5–6.0 mm (1.7–3.81 mm in Australian species). Rostrum in repose not reaching middle of mesocoxal length. Scutellar shield completely covered by elytra. Mesothoracic receptacle deep, posteriorly closed. Metanepisternum completely absent. Metathoracic spiracle externally on side of metaventrite. Elytra with 9 striae (sometimes superficially effaced). Tarsal claws minute. Body largely unclothed (densely squamose in *Trigonopterus squamosus* Lea and *Trigonopterus striatipennis* Lea). Metafemur in Australian species without stridulatory patch. For additional information, see van de Kamp et al. (2015) and http://species-id.net/wiki/Trigonopterus.

### Descriptions of the species

#### 1. Trigonopterus aequalis

Taxon classificationAnimaliaColeopteraCurculionidae

(Pascoe)

Idotasia aequalis Pascoe, 1872: 100.
Trigonopterus aequalis (Pascoe): Pullen et al. 2014: 271.

##### Diagnostic description.

**Figure 1.**
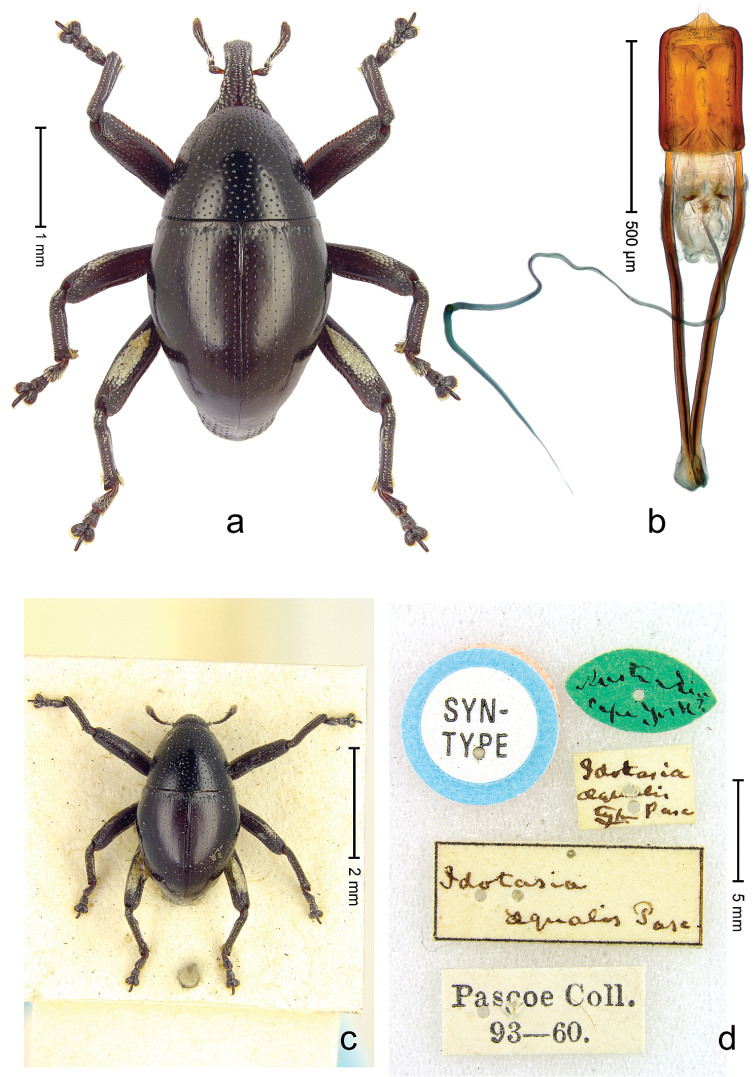
*Trigonopterus aequalis* (Pascoe), lectotype; **a** Habitus **b** Penis **c** as mounted originally **d** original labels.

Lectotype (Fig. 1a). Length 3.50 mm. Color black, legs tending to deep ferruginous. Body subovate, almost without constriction between pronotum and elytron; in profile evenly convex. Rostrum with median ridge and pair of submedian ridges; intervening furrows with rows of silvery scales; apical 1/3 rugose-punctate. Eyes with dorsal margin weakly carinate, bordered by furrow. Forehead coarsely punctate. Pronotum with disk punctate; sides foveate; interspaces microreticulate; base medially hardly extended towards elytral suture. Elytra with striae marked by distinct rows of minute punctures, interspaces weakly microreticulate; along base and humeri with row of large punctures; apex densely punctate. Legs. Femora microreticulate, punctate. Metafemur dorsally with elongate patch of dense white scales; posterior surface with ventral edge rimmed by costa and row of scales, with longitudinal furrow containing row of scales parallel to indistinct dorsoposterior edge. Mesotibia apically with uncus and larger premucro approximate at base but not fused, widely diverging. Metatibia apically with uncus and small angular premucro. Abdominal ventrite 2 swollen, with posterior edge projecting, medially forming common cavity with ventrite 1; ventrite 5 dull, microreticulate, punctate, almost flat, with weak impression. Penis (Fig. 1b) with sides of body subparallel; apex with median triangular extension somewhat confluent with outline of apex; transfer apparatus short, dentiform, bordered by indistinct sclerites; ductus ejaculatorius without bulbus.

##### Material examined.

Type specimens. Male, lectotype by present designation (Fig. 1) (BMNH): Queensland, Cape York ? (labels Fig. 1d), ARC4079 (PCR failed). Other specimens (ANIC): 1 ex, Warrah [S Tamworth, leg. W.W. Froggatt].

##### Distribution.

New South Wales: Tamworth.

##### Notes.

Pascoe (1872) did not designate a holotype nor specify the number of specimens examined but gave two localities, “Cape York” and “Rockhampton”. Only the syntype from the first locality could be located in the BMNH. Presumably the missing syntype from Rockhampton represents a different species. A lectotype is designated here to achieve stability of nomenclature.

The question mark behind the name of the type locality (“Cape York ?”) suggests that there was doubt about its validity already in Pascoe’s times. This is supported by the fact that we could only examine one additional specimen, identified by Lea, from the village of Warrah, south of Tamworth in New South Wales. Additional field work should verify the occurrence of the species in this area.

#### 2. Trigonopterus albidosparsus

Taxon classificationAnimaliaColeopteraCurculionidae

(Lea)

Idotasia albidosparsa Lea, 1913: 611.
Trigonopterus albidosparsa (Lea), incorrect subsequent spelling: Zimmerman 1992: 376.
Trigonopterus albidosparsus (Lea): Pullen et al. 2014: 271.

##### Diagnostic description.

**Figure 2:**
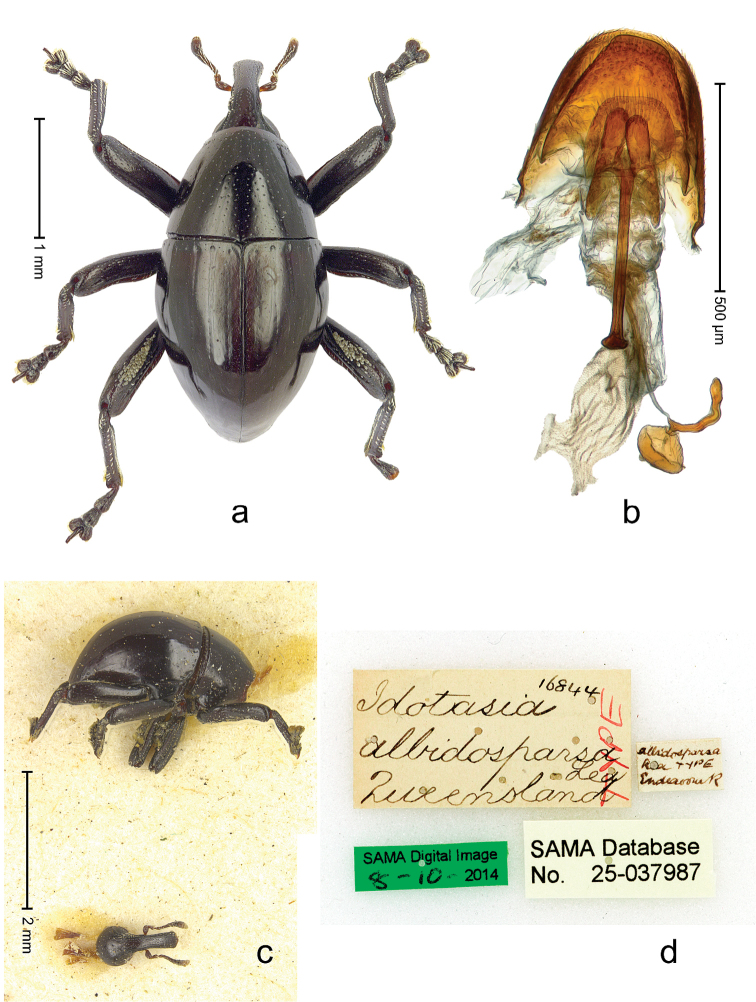

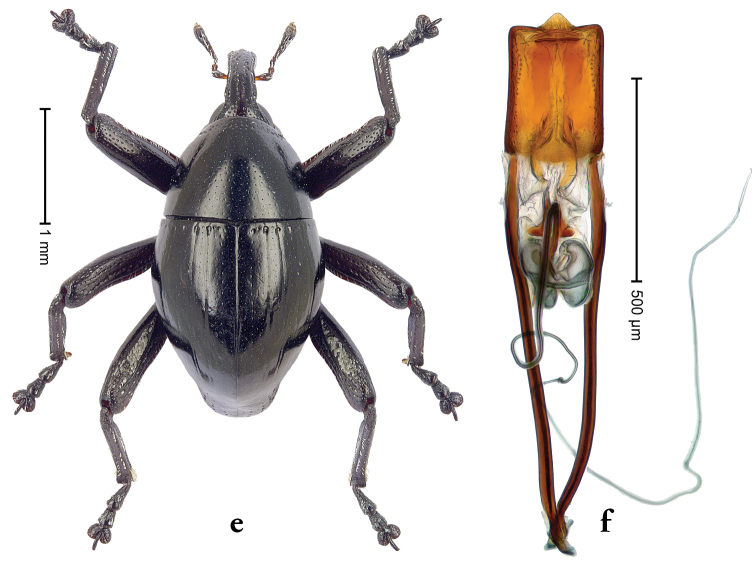
*Trigonopterus albidosparsus* (Lea), female lectotype; **a** Habitus **b** Terminalia **c** as mounted originally **d** original labels. *Trigonopterus albidosparsus* (Lea), male; **e** Habitus **f** penis.

Male (ARC3695; Fig. 2e). Length 2.73 mm. Color black. Body subovate, almost without constriction between pronotum and elytron; in profile evenly convex. Rostrum with median costa and pair of submedian costae; intervening furrows with rows of partly abraded scales; apical 1/3 rugose-punctate. Eyes with dorsal margin bordered by furrow. Forehead sparsely punctate. Pronotum with disk subglabrous, with minute punctures; sides foveate; interspaces not microreticulate; base medially hardly extended towards elytral suture. Elytra subglabrous, striae marked by very shallow lines, without punctures; along base and humeri with row of large punctures; apex with scattered shallow punctures. Legs. Femora microreticulate, punctate. Metafemur dorsally with elongate patch of dense silvery scales; posterior surface with pair of longitudinal furrows containing rows of scales parallel to ventral and dorsal edge; dorsoposterior edge distinct. Mesotibia apically with widened uncus only, premucro absent. Metatibia apically with uncus only, premucro not visible in lateral aspect, possibly transformed into small process on posterior tibial face near tarsal insertion. Abdominal ventrite 2 swollen, posterior edge projecting and with submedian pair of denticles, medially forming shallow cavity with ventrite 1; ventrite 5 dull, microreticulate, punctate, with transversely ovate cavity. Penis (Fig. 2f) with sides of body subparallel; apex with distinct median triangular extension; transfer apparatus long, spiniform, apically bordered by pair of L-shaped sclerites; ductus ejaculatorius without bulbus. **Female lectotype** (Fig. 2a–d). As male except: length 2.63 mm. Rostrum dorsally subglabrous, densely punctate with small punctures. Abdominal ventrites 1 and 2 medially flat; posterior edge of ventrite 2 simple, without pair of denticles; abdominal ventrite 5 flat. **Intraspecific variation.** Length 2.26–2.73 mm. Mesotibia apically with large uncus and much smaller premucro.

##### Material examined.

Type specimens. Female, lectotype by present designation (SAMA): Queensland, Endeavour River (labels Fig. 2d), ARC4033 (PCR failed). Other specimens (ANIC, QMBA, SMNK): 36 exx, ARC3695 (EMBL # LN888180), ARC3696 (EMBL # LN888181), ARC3697 (EMBL # LN888182), Cooktown, Mt. Cook N.P., S15°28.648', E145°15.793', to S15°29.252', E145°15.992', 63-324 m, 24-IV-2014; 31 exx, Cooktown, Mt. Cook N.P., S15°28.648', E145°15.793', to S15°29.252', E145°15.992', 63–245 m, 23-IV-2014; 10 exx, Mt. Cook N.P., S15°29', E145°16', 11-12-X-1980; 1 ex, 1 km SE Mt. Cook, S15°30', E145°16', 13-X-1980.

##### Distribution.

Queensland: Cooktown.

##### Biology.

Beaten from foliage in rainforest.

##### Notes.

Lea (1913) did not designate a holotype in the original description, which is based on three syntypes. One syntype labelled “type” could be examined and is here designated as lectotype. The species appears to be confined to the Cooktown area. Numerous specimens from other localities of coastal Queensland, including some identified as *Trigonopterus albidosparsa* by Lea, belong to different, closely related species.

#### 3 . Trigonopterus allaetus

Taxon classificationAnimaliaColeopteraCurculionidae

Riedel sp. n.

http://zoobank.org/9C9576E3-6866-4523-A726-5AF39807037B

##### Diagnostic description.

**Figure 3.**
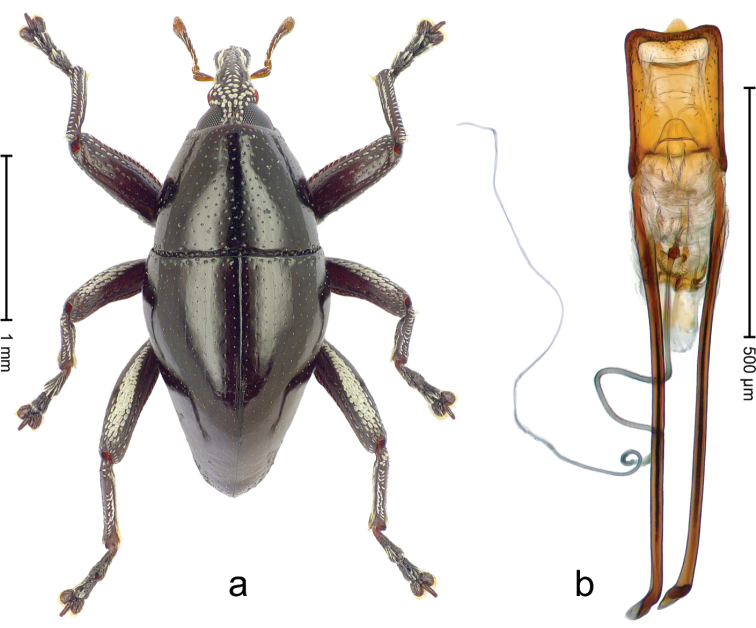
*Trigonopterus allaetus* sp. n., holotype; **a** Habitus **b** Penis.

Holotype (Fig. 3a). Length 2.34 mm. Color black, antenna ferruginous. Body elongate-ovate, with weak constriction between pronotum and elytron; in profile evenly convex. Rostrum with median costa and pair of fine submedian ridges; intervening furrows with rows of white erect scales. Eyes large, in dorsal position, medially approximate. Pronotum with disk subglabrous, densely punctate with minute punctures; sides more densely punctate with larger punctures. Elytra subglabrous, with sparse minute punctures; along base and humeri with row of larger punctures. Legs. Meso- and metafemur dorsally with narrow band of white scales; metafemur with distinct dorsoposterior edge. Tibial apex with uncus and minute premucro. Abdominal ventrites 1-2 and ventrite 5 medially concave. Abdominal venter medially subglabrous, laterally with sparse white scales. Penis (Fig. 3b) with sides of body subparallel; apex with median acute extension; transfer apparatus short, dentiform; ductus ejaculatorius without bulbus. **Intraspecific variation.** Length 2.16–2.68 mm. Female rostrum subglabrous, sparsely setose, with submedian rows of punctures; base squamose. Female abdominal ventrites 5 flat.

##### Material examined.

Holotype (ANIC): ARC4240 (PCR failed), Queensland, 8 km E Mt. Tozer, S12°45', E143°17', 08-VII-1986. Paratypes (ANIC, SMNK): Queensland: 1 ex, 3 km ENE Mt. Tozer, S12°45', E143°14', swept from u´growth, 28-VI-04-VII-1986; 6 exx (ARC4241, PCR failed), 9 km ENE of Mt. Tozer, S12°43', E143°17', beating rainforest vegetation, 05-10-VII-1986; 3 exx, 11 km ENE of Mt. Tozer, S12°43', E143°18', 11-16-VII-1986.

##### Distribution.

Queensland: Iron Range.

##### Biology.

Swept and beaten from rainforest vegetation.

##### Etymology.

This epithet is a combination of the Latin prefix *ad*- (next to; near) and the specific epithet of *Trigonopterus laetus* (Lea), a closely related species.

#### 4. Trigonopterus athertonensis

Taxon classificationAnimaliaColeopteraCurculionidae

Riedel sp. n.

http://zoobank.org/DC95607E-F0F1-4532-A480-034564653309

##### Diagnostic description.

**Figure 4.**
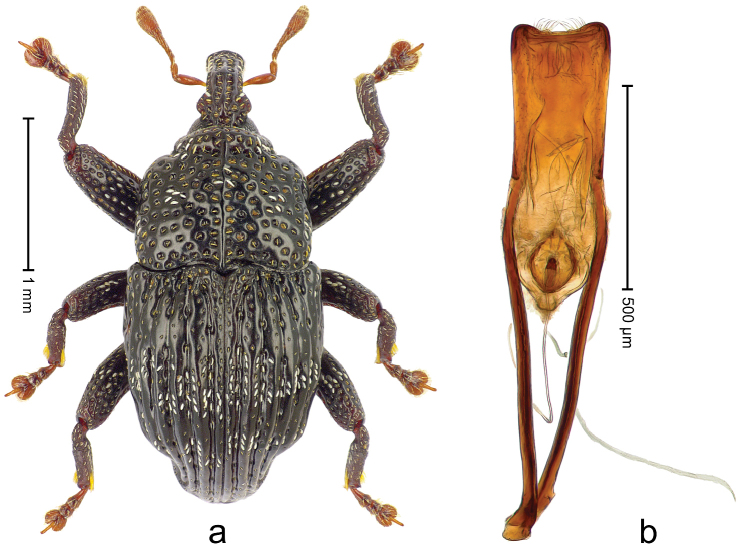
*Trigonopterus athertonensis* Riedel, sp. n., holotype; **a** Habitus **b** Penis.

Holotype (Fig. 4a). Length 2.45 mm. Color black; antenna and tarsi ferruginous. Body elongate, with distinct constriction between pronotum and elytron; in profile convex. Rostrum with median costa and pair of submedian costae ending in apical third; intervening furrows with rows of coarse punctures each containing one mesad directed narrow seta; base dorsally protruding, projecting from profile subangularly; epistome posteriorly with transverse ridge. Forehead coarsely punctate-rugose. Pronotum with subapical constriction; disk coarsely punctate; with median costa; punctures each containing one seta, few with white scale. Elytra with striae deeply incised, containing few coarse punctures; intervals costate, sparsely punctate, in basal third partly transversely confluent; with indistinct transverse bands of sparse white scales; base markedly bisinuate. Legs. Femora densely punctate, with transverse band of sparse white scales. Profemur with subbasal callus anteriorly projecting. Tibiae subbasally with dorsal angulation; metatibia with suprauncal denticle. Abdominal ventrites 1 medially concave; abdominal ventrite 2 swollen, especially laterally; ventrite 5 in basal half concave, coarsely punctate. Penis (Fig. 4b) with sides of body weakly diverging to subtruncate apex; transfer apparatus compact, subrotund; ductus ejaculatorius without bulbus. **Intraspecific variation.** Length 2.45–2.68 mm.

##### Material examined.

Holotype (ANIC): ARC4041 (PCR failed), Queensland, Mt. Fisher, 7 km SW Millaa Millaa, S17°34', E145°34', 1100 m, rainforest, litter, Q.M. Berlesate No. 409, 27-IV-1982. Paratypes (ANIC, QMBA, SMNK): Queensland: 1 ex, same data as holotype; 2 exx, Mt. Fisher, 7 km SW Millaa Millaa, S17°34', E145°34', 1050 m, rainforest, litter, Q.M. Berlesate No. 412, 27-IV-1982; 1 ex, Mt Fisher, summit, 17°34'S, 145°33'E, rainforest, 1360 m, sieved litter, Berlesate 991, 08-II-1999.

##### Distribution.

Queensland: Mt. Fisher.

##### Biology.

Sifted from leaf litter in primary forest.

##### Etymology.

This epithet is an adjective based on the name of the Atherton Tablelands, where the species occurs.

#### 5. Trigonopterus australinasutus

Taxon classificationAnimaliaColeopteraCurculionidae

Riedel sp. n.

http://zoobank.org/77D735C6-FD14-48D5-BFB0-C5ACA3E7FA7D

##### Diagnostic description.

**Figure 5.**
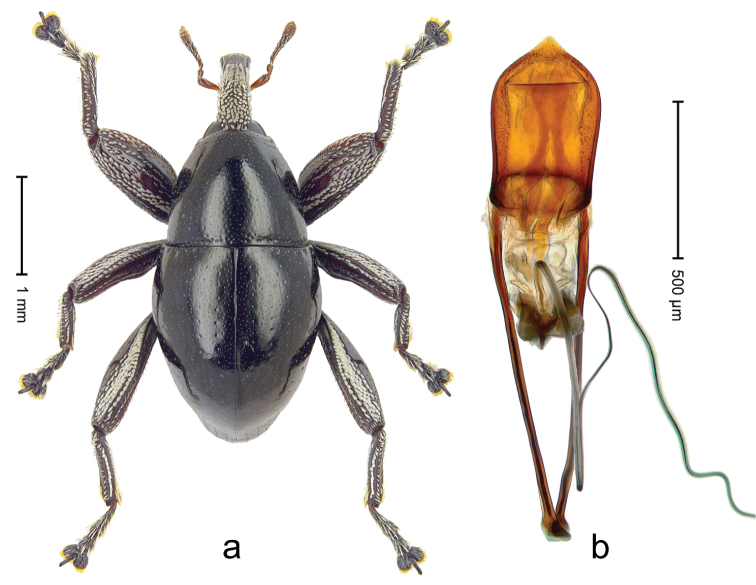
*Trigonopterus australinasutus* sp. n., holotype; **a** Habitus **b** Penis.

Holotype (Fig. 5a). Length 3.28 mm. Color black; legs deep ferruginous to black; antenna lighter ferruginous. Body subovate; in dorsal aspect and in profile with weak constriction between pronotum and elytron. Rostrum in basal half dorsally markedly swollen, punctate-reticulate, densely squamose with white erect scales; subapical third subglabrous, weakly punctate, sparsely setose. Eyes medially approximate. Pronotum subglabrous, with minute punctures; laterally punctures somewhat larger; evenly rounded towards sides; in front of procoxa with acute process. Elytra subglabrous with minute punctures; striae indistinct; basal margin straight, towards sides bordered by row of moderately deep punctures. Femora with anteroventral ridge distinct, rounded basally; at middle with small tooth. Mesofemur and metafemur dorsally densely squamose with white scales; metafemur with distinct dorsoposterior edge. Abdominal ventrites 1-2 medially concave. Abdominal venter medially subglabrous, laterally with sparse white scales. Penis (Fig. 5b) apically subangulate, weakly pointed; transfer apparatus beak-shaped, pointed, directed basad; ductus ejaculatorius without bulbus. **Intraspecific variation.** Length 2.98–3.47 mm. Female rostrum in apical 2/3 dorsally flattened, subglabrous, sparsely punctate; basally swollen, with erect white scales.

##### Material examined.

Holotype (ANIC): ARC4238 (PCR failed), Queensland, 11 km ENE of Mt. Tozer, S12°43', E143°18', 11-16-VII-1986. Paratypes (ANIC, QMBA, SMNK): Queensland: 2 exx, same data as holotype; 5 exx, ARC4239 (PCR failed), 3 km ENE Mt. Tozer, S12°44', E143°14', 28-VI-04-VII-1986; 1 ex, 8 km E by N of Mt. Tozer, S12°44', E143°17', beating rainforest vegetation, 07-VII-1986; 3 exx, 9 km ENE of Mt. Tozer, S12°43', E143°17', 05-10-VII-1986; 1 ex, Claudie R., nr. Iron Rg., under bark, rotten logs, 19-25-VII-1978; Gordon Creek area, Claudie Riv. District, 23-I-1982.

##### Distribution.

Queensland: Iron Range.

##### Biology.

Swept and beaten from rainforest vegetation.

##### Etymology.

This epithet is a combination of the Latin adjective *australis* (southern) and the specific epithet of *Trigonopterus nasutus* (Pascoe), also an adjective.

##### Notes.

This species is closely related to *Trigonopterus nasutus* (Pascoe) and *Trigonopterus gibbirostris* (Faust) from New Guinea. From the former it can be distinguished by a longer and spiniform transfer apparatus, from the latter by its medially pointed apex of the penis.

#### 6. Trigonopterus australis

Taxon classificationAnimaliaColeopteraCurculionidae

Riedel sp. n.

http://zoobank.org/535F820A-1CB9-4649-BE2E-303DC40908D2

##### Diagnostic description.

**Figure 6.**
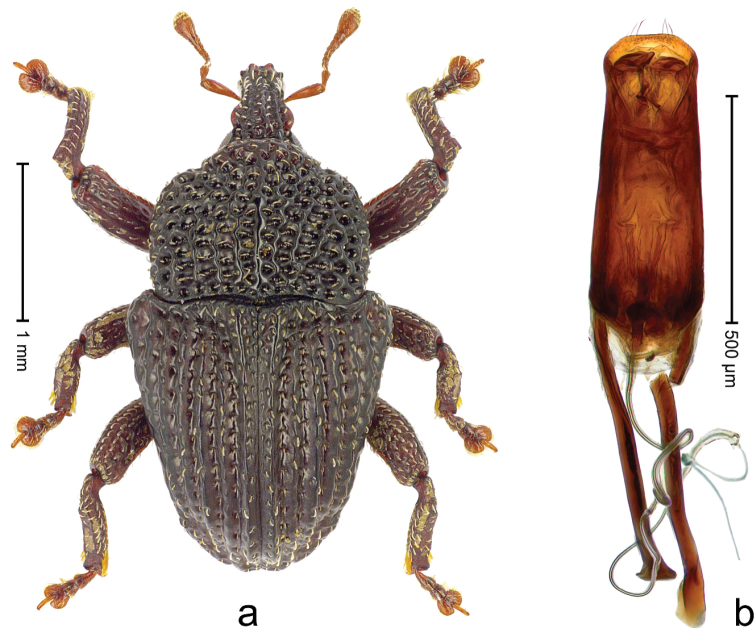
*Trigonopterus australis* sp. n., holotype; **a** Habitus **b** Penis.

Holotype (Fig. 6a). Length 2.78 mm. Color black; antenna and legs ferruginous. Body subovate, in dorsal aspect and in profile with marked constriction between pronotum and elytron. Rostrum punctate-scabrous, in basal third with median ridge and pair of submedian ridges; in front of antennal insertion with median bifid protrusion; punctures containing upcurved narrow scales; epistome posteriorly with curved ridge bearing 4 denticles. Forehead coarsely punctate-rugose. Pronotum with sides subparallel, anteriorly abruptly rounded to indistinct subapical constriction; irregularly foveate-reticulate; each fovea containing one inconspicuous seta; interspaces forming irregular, mainly longitudinal ridges. Elytra converging from humeri to apex; base bisinuate; striae deeply impressed, with coarse punctures; intervals carinate to costate; with sparse subrecumbent scales; sutural interval prominent, near base markedly swollen. Legs. Femora punctate-rugose, with sparse suberect scales. Tibiae subbasally with dorsal angulation; metatibia subapically with suprauncal angulation. Metaventrite subglabrous except deep median furrow. Abdominal ventrite 1 concave; abdominal ventrite 2 posteriorly transversely costate. Penis (Fig. 6b) with sides of body weakly converging from base to subtruncate apex; in profile ventrally with marked subapical swelling; transfer apparatus short, dentiform; ductus ejaculatorius without bulbus. **Intraspecific variation.** Length 1.92–3.34 mm. Body usually covered with more or less thick soil incrustations removed in holotype. Female body slender. Female rostrum dorsally somewhat flattened, without protrusion; in basal half with median costa and pair of submedian costae; epistome simple.

##### Material examined.

Holotype (QMBA): ARC3895 (PCR failed), Queensland, West Claudie R., Iron Range, S12°45', E143°14', sieved litter, Berlesate No. 693, 50 m, 05-XII-1985. Paratypes (ANIC, SMNK): Queensland: 27 exx, 11 km ENE of Mt. Tozer, S12°43', E143°18', rainforest litter, Berlesate ANIC 1065, 11-16-VII-1986; 2 exx, 9 km ENE of Mt. Tozer, S12°43', E143°17', open forest litter, Berlesate ANIC 1061, 05-10-VII-1986; 1 ex, 3 km ENE of Mt. Tozer, S12°44', E143°14', flight intercept trap, rainforest, 28-VI-16-VII-1986; 1 ex, 3 km ENE of Mt. Tozer, S12°44', E143°14', Berlesate ANIC 1052 rainforest litter, 01-04- VII-1986; 3 exx, Claudie R. nr. Iron Rg., 19-25-VII-1978; 3 exx, Iron Ra., S12°45', E143°14', Berlesate ANIC 309, rainforest, 14-VI-1971; 1 ex, Iron Ra., S12°43', E143°48', Berlesate ANIC 308, rainforest, 15-VI-1971; 3 exx, ARC4042 (PCR failed), McIlwraith Range, 8km WbyN of Bald Hill, upper Leo creek site, S13°45', E143°22', berlesate ANIC 1117, leaf litter, closed forest, 500 m, 27-VI-12-VII-1989; 6 exx, McIlwraith Range, 8km WbyN of Bald Hill, mango tree site, S13°45', E143°22', berlesate ANIC 1118, leaf litter, closed forest, 500 m, 27-VI-12-VII-1989; 14 exx, McIlwraith Range, 11km WbyN of Bald Hill, search party campsite, S13°44', E143°20', berlesate ANIC 1107, leaf litter, closed forest, 520 m, 27-VI-12-VII-1989; 1 ex, McIlwraith Range, 15km WNW of Bald Hill, interface site, S13°43', E143°19', berlesate ANIC 1122, leaf litter, monsoon forest with *Casuarina* & *Acacia*, 500 m, 27-VI-12-VII-1989.

##### Distribution.

Queensland: Mc Ilwraith Range, Iron Range.

##### Biology.

Sifted from leaf litter in primary forest.

##### Etymology.

This epithet is the Latin adjective *australis* (southern) and refers to the continent formerly known as “Terra Australis”, i.e. Australia.

#### 7. Trigonopterus bisignatus

Taxon classificationAnimaliaColeopteraCurculionidae

Riedel sp. n.

http://zoobank.org/5A37941C-DBC6-4012-BBB7-F22D8B6C910D

##### Diagnostic description.

**Figure 7.**
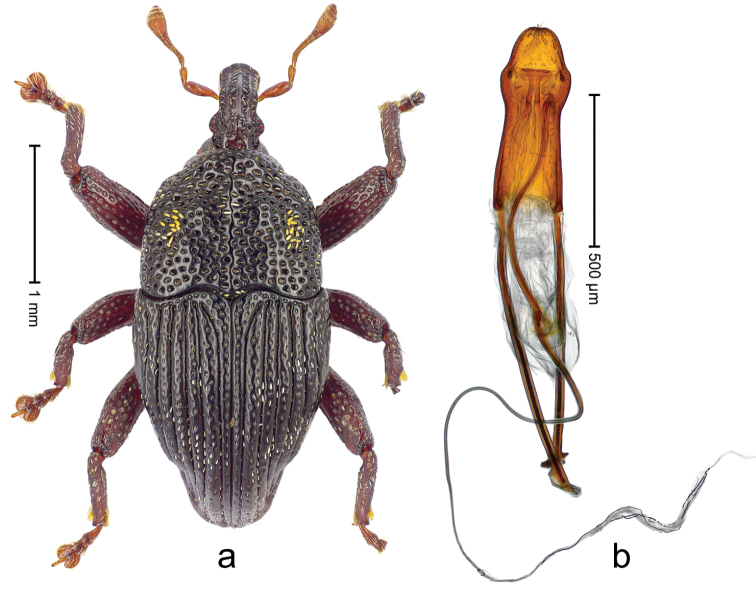
*Trigonopterus bisignatus* sp. n., holotype; **a** Habitus **b** Penis.

Holotype (Fig. 7a). Length 2.73 mm. Color black; antenna and legs ferruginous. Body subovate, with shallow constriction between pronotum and elytron; in profile convex. Rostrum with median ridge and pair of submedian ridges ending in apical third; intervening furrows with rows of coarse punctures each containing one mesad directed seta; base dorsally protruding, markedly projecting from profile subangularly; epistome posteriorly with curved ridge bearing 4 low denticles. Forehead coarsely punctate-rugose. Pronotum with indistinct subapical constriction; disk coarsely punctate-reticulate; with median costa; near middle with pair of very weak swellings, further laterad with clusters of yellow recumbent scales. Elytra with striae deeply incised; intervals costate, microreticulate, with shallow punctures and few scattered recumbent scales; base markedly bisinuate. Legs. Femora densely punctate. Profemur with subbasal callus anteriorly projecting. Tibiae subbasally with dorsal angulation. Abdominal ventrites 1-2 laterally swollen, medially forming common cavity; ventrite 5 punctate, flat. Penis (Fig. 7b) with sides of body weakly converging, at middle with constriction, widening to subtriangular apex; transfer apparatus flagelliform, ca. 1.5× longer than body of penis; ductus ejaculatorius near insertion to transfer apparatus sclerotized, without bulbus. **Intraspecific variation.** Length 2.73–3.03 mm.

##### Material examined.

Holotype (QMBA): ARC3752 (EMBL # LN888232), Queensland, Daintree N.P., NW Mossman, Manjal Jimalji (Devils Thumb) trail, S16°23.571', E145°19.058', sample 2, 377 m, 20-IV-2014. Paratypes (ANIC): Queensland: 1 ex, ARC4053 (PCR failed), Windsor Tableland, 35 km NW Mt. Carbine, 1050 m, rainforest, sieved litter, Berlesate No. 393, 16-IV-1982; 2 exx, ARC4050 (PCR failed), Mossman Gorge N.P., 6 km SW of Mossman, rainforest leaf litter, 50 m, 11-VII-1982.

##### Distribution.

Queensland: Daintree N.P., Windsor Tableland.

##### Biology.

Sifted from leaf litter in primary forest.

##### Etymology.

This epithet is a combination of the Latin prefix *bi*- (two) and the participle *signatus* (marked) and refers to the pair of squamose patches on the pronotum.

##### Notes.

*Trigonopterus bisignatus* Riedel, sp. n. was coded as “*Trigonopterus* sp. 560”.

#### 8. Trigonopterus bisinuatus

Taxon classificationAnimaliaColeopteraCurculionidae

Riedel sp. n.

http://zoobank.org/CBB64820-13B8-41E4-A1D8-0541C087704C

##### Diagnostic description.

**Figure 8.**
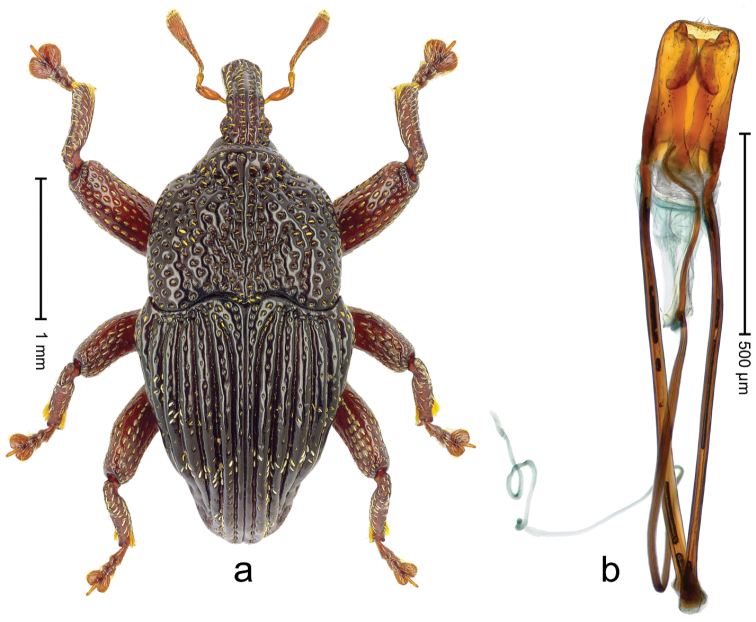
*Trigonopterus bisinuatus* sp. n., holotype; **a** Habitus **b** Penis.

Holotype (Fig. 8a). Length 2.88 mm. Color black; antenna and legs ferruginous. Body subovate, with shallow constriction between pronotum and elytron; in profile convex. Rostrum with median ridge and pair of submedian ridges ending in apical third; intervening furrows with rows of coarse punctures each containing one mesad directed seta; base dorsally protruding, projecting from profile subangularly; epistome posteriorly with 4 low denticles. Forehead coarsely punctate-rugose. Pronotum with subapical constriction; disk coarsely punctate-rugose; with median costa; submedially punctures confluent forming irregular longitudinal furrows and wrinkles, near middle sparing pair of weak swellings of irregular outline. Elytra with striae deeply incised; intervals costate, sparsely punctate, with few scattered recumbent white scales; base markedly bisinuate. Legs. Femora densely punctate. Profemur with subbasal callus anteriorly projecting. Tibiae subbasally with dorsal angulation; metatibia subapically with suprauncal denticle. Abdominal ventrites 1-2 laterally swollen, medially forming common cavity; ventrite 5 punctate, weakly concave. Penis (Fig. 8b) with sides of body subparallel, apex rounded; behind ostium with pair of sclerites; endophallus with pair of elongate sclerites; transfer apparatus flagelliform, ca. 1.6× longer than body of penis; ductus ejaculatorius near insertion to transfer apparatus sclerotized, this portion longer and thicker than flagellum; without bulbus. **Intraspecific variation.** Length 2.60–2.90 mm. Female body more slender. Female rostrum dorsally somewhat flattened; median costa and pair of submedian costae subglabrous; epistome simple. Female abdominal ventrites 1-2 medially flat; ventrite 5 coarsely punctate, basally swollen, apically flat.

##### Material examined.

Holotype (QMBA): ARC3736 (EMBL # LN888218), Queensland, Garradunga, Polly Ck., N Innisfail, Hasenpusch property, S17°27.458', E146°01.227', sample 4, 82 m, 16-IV-2014. Paratypes (ANIC, QMBA, SMNK): Queensland: 3 exx, ARC3737 (EMBL # LN888219), ARC3738 (EMBL # LN888220), ARC3739 (EMBL # LN888221), same data as holotype; 7 exx, Garradunga, Polly Ck., N Innisfail, Hasenpusch property, S17°27.388', E146°01.200', sample 1, 70 m, 16-IV-2014; 7 exx, Garradunga, Polly Ck., N Innisfail, Hasenpusch property, S17°27.306', E146°01.214', sample 2, 103 m, 16-IV-2014; 3 exx, Garradunga, Polly Ck., N Innisfail, Hasenpusch property, S17°27.252', E146°01.222', sample 3, 105 m, 16-IV-2014; 22 exx, Garradunga, Polly Ck., N Innisfail, Hasenpusch property, S17°27.458', E146°01.227', sample 4, 82 m, 16-IV-2014; 1 ex Stone Ck (Hasenpusch), 01-XI-1995, 06-II-1996, 100 m, pitfall traps; 4 exx, ARC3719 (EMBL # LN888201), ARC3720 (EMBL # LN888202), ARC3721 (EMBL # LN888203), ARC3722 (EMBL # LN888204), Wooroonooran N.P., Palmerston Highway, K-tree-road, sample 3, 428 m, S17°36.510', E145°46.074', 04-IV-2014; 1 ex, ARC3718 (EMBL # LN888200), Danbulla N.P., Robson´s Creek, S17°07.14', E145°37.92', 700 m, 09-12-IV-2014, ex dung pitfall trap; 3 exx, Graham Range, 550 m, S17°17', E145°58', 01-XI-8-XII-1995, pitfall traps; 3 exx, Graham Range, 01-XI-1995, Berlesate 895, S17°17', E145°58', rainforest, 550 m; 1 ex, Graham Range, 08-XII-1995, Berlesate 901, S17°17', E145°58', rainforest, 550 m; 3 exx, North Bell Peak, 22-XI-1990, Berlesate 845, S17°06', E145°52', rainforest, 600 m; 1 ex, Kauri Creek, 2 km E, S17°08', E145°37', 10-11-II-1999, 680 m, rainforest, dung pitfall, 2191; 1 ex, Mt. Murray Prior, 30-X-1995, Berlesate 894, S16°56', E145°51', rainforest, 770 m, sieved litter; 1 ex ARC4049 (PCR failed), 4 km E Lake Barrine, S17°16', E145°41', ANIC Berlesate 352, rainforest, 01-VII-1971; 1 ex, Eacham N.P., S17°18', E145°37', ANIC Berlesate 435, rainforest, 20-II-1973; 1 ex, Eacham N.P., S17°18', E145°37', 760 m, ANIC Berlesate 437, rainforest, 19-II-1973; 1 ex, Eacham N.P., S17°18', E145°37', 760 m, ANIC Berlesate 484, rainforest, 23-III-1973; 2 exx, 3.2 km SW Little Mulgrave, ANIC Berlesate 263, rainforest, 25-II-1970; 1 ex, Barrine N.P., S17°16', E145°38', ANIC Berlesate 486, 21-III-1975.

##### Distribution.

Queensland: Atherton Tablelands, Danbulla N.P., Garradunga, Graham Range, Kauri Creek, Mt. Murray Prior, North Bell Peak, Wooroonooran N.P.

##### Biology.

Sifted from leaf litter in primary forest; occasionally found in pitfall traps.

##### Etymology.

This epithet is a combination of the Latin prefix *bi*- (two) and the participle *sinuatus* (curved) and refers to the outline of the elytral base.

##### Notes.

*Trigonopterus bisinuatus* Riedel, sp. n. was coded as “*Trigonopterus* sp. 561”.

#### 9. Trigonopterus boolbunensis

Taxon classificationAnimaliaColeopteraCurculionidae

Riedel sp. n.

http://zoobank.org/4FD5DC0C-580E-4F35-B729-7730A23089B4

##### Diagnostic description.

**Figure 9.**
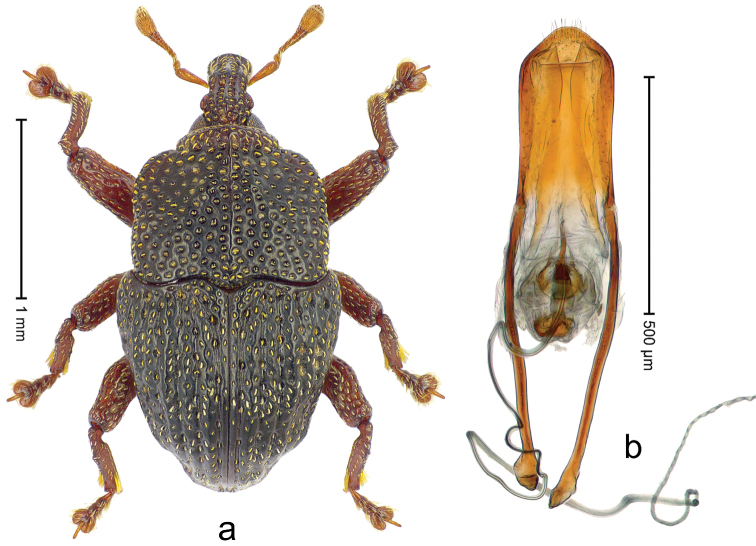
*Trigonopterus boolbunensis* sp. n., holotype; **a** Habitus **b** Penis.

Holotype (Fig. 9a). Length 2.10 mm. Color of head, legs, and sides of pronotum ferruginous; remainder black. Body subovate, with distinct constriction between pronotum and elytron; in profile convex. Rostrum with median ridge and pair of submedian ridges ending in apical third; intervening furrows with rows of mesad directed setae; base dorsally protruding, projecting from profile; epistome posteriorly with transverse ridge. Forehead coarsely punctate-rugose. Pronotum subquadrate; sides subparallel; with distinct subapical constriction; coarsely punctate; each puncture containing one small seta, laterally each with one yellowish scale; with indistinct median ridge. Elytral striae weakly incised, with sparse rows of punctures; intervals costate, with small punctures containing small recumbent cream-colored scales; base bisinuate. Legs. Femora rugose-punctate; with scattered, narrow, cream-colored scales. Tibiae subbasally with dorsal angulation. Abdominal ventrites 1-2 laterally swollen, medially forming common cavity, with coarse punctures; ventrite 5 punctate, weakly concave. Penis (Fig. 9b) with sides of body subparallel; apex subangulate; endophallus with anchor-shaped basal sclerite; transfer apparatus spiniform; ductus ejaculatorius without bulbus.

##### Material examined.

Holotype (QMBA): ARC3894 (PCR failed), Queensland, Mt. Boolbun South, S15°57', E145°08', 850-1000 m, rainforest, leaf litter, Berlesate 896, 06-XI-1995.

##### Distribution.

Queensland: Mt. Boolbun.

##### Biology.

Sifted from leaf litter in primary forest.

##### Etymology.

This epithet is an adjective and refers to the name of the type locality, Mt. Boolbun.

#### 10. Trigonopterus cooktownensis

Taxon classificationAnimaliaColeopteraCurculionidae

Riedel sp. n.

http://zoobank.org/2728105D-13D1-41AC-856D-0B65DD4898A2

##### Diagnostic description.

**Figure 10.**
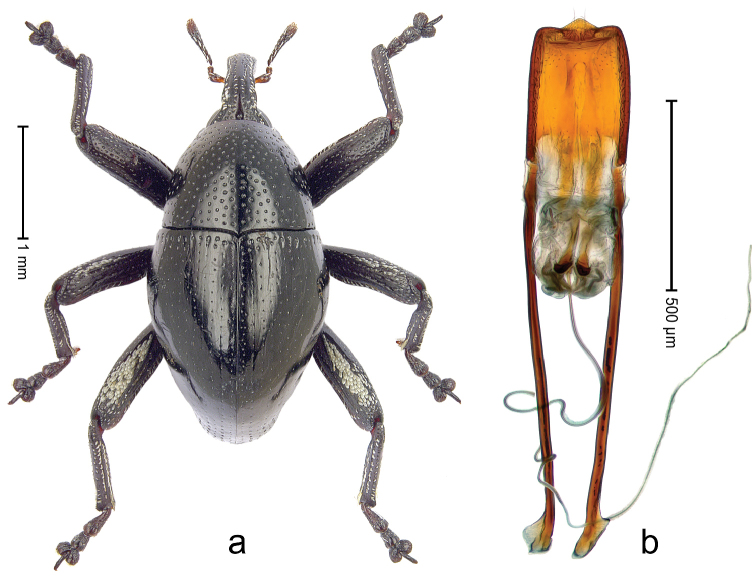
*Trigonopterus cooktownensis* sp. n., holotype; **a** Habitus **b** Penis.

Holotype (Fig. 10a). Length 2.95 mm. Color black. Body subovate, almost without constriction between pronotum and elytron; in profile evenly convex. Rostrum with median costa and pair of submedian costae dorsally flattened; intervening furrows with rows of silvery scales; apical 1/3 rugose-punctate. Eyes with dorsal margin bordered by furrow. Forehead with sparse coarse punctures. Pronotum with disk punctate; sides with punctures slightly larger; interspaces not microreticulate; base slightly extended towards elytral suture. Elytra with striae marked by rows of minute punctures; along base and humeri with row of large punctures; apex with dense rows of small shallow punctures. Legs. Femora microreticulate, punctate. Metafemur dorsally with elongate patch of dense silvery scales; posterior surface with pair of longitudinal furrows containing rows of scales parallel to ventral and dorsal edge; dorsoposterior edge indistinct. Mesotibia apically with uncus and premucro largely fused, with shallow incision at apex. Metatibia apically with uncus and distinct premucro. Abdominal ventrite 2 swollen, with posterior edge projecting, medially forming common cavity with ventrite 1; ventrite 5 concave, dull, microreticulate, punctate. Penis (Fig. 9b) with sides of body subparallel, weakly converging; apex with median triangular extension confluent with outline of apex; transfer apparatus short, dentiform, apically bordered by pair of L-shaped sclerites; ductus ejaculatorius without bulbus.

##### Material examined.

Holotype (QMBA): ARC3698 (EMBL # LN888183), Queensland, Cooktown, Mt. Cook N.P., S15°28.648', E145°15.793', to S15°29.252', E145°15.992', 63-245 m, 23-IV-2014. Paratypes (ANIC, SMNK): Queensland: 1 ex, Cooktown, Mt. Cook N.P., S15°29', E145°16', 11-12-X-1980; 1 ex, Cooktown, Mt. Cook N.P., S15°28.648', E145°15.793', to S15°29.252', E145°15.992', 63-324 m, 24-IV-2014.

##### Distribution.

Queensland (Mt. Cook).

##### Biology.

Beaten from foliage of *Acacia*-dominated forest.

##### Etymology.

This epithet is an adjective based on the name of the type locality, Cooktown.

##### Notes.

*Trigonopterus cooktownensis* Riedel, sp. n. was coded as “*Trigonopterus* sp. 566”. It occurs syntopically with *Trigonopterus albidosparsus* Lea.

#### 11. Trigonopterus daintreensis

Taxon classificationAnimaliaColeopteraCurculionidae

Riedel sp. n.

http://zoobank.org/96C266B2-62A1-4581-8551-97784D4A0279

##### Diagnostic description.

**Figure 11.**
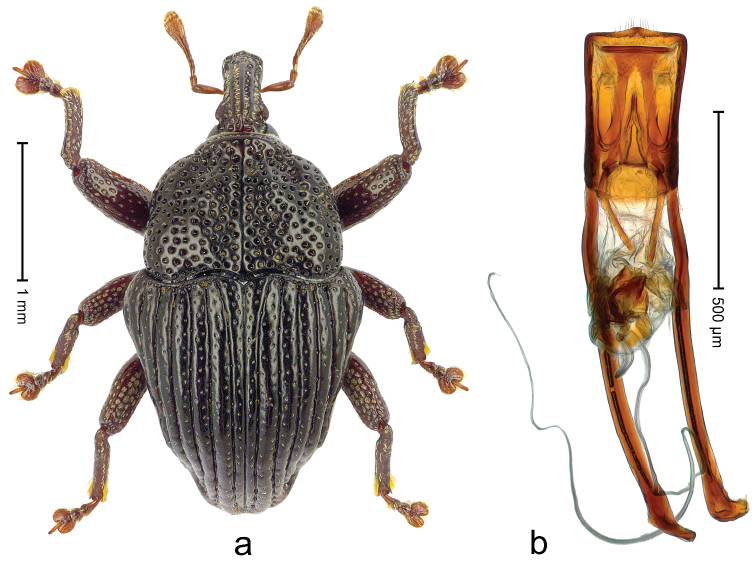
*Trigonopterus daintreensis* sp. n., holotype; **a** Habitus **b** Penis.

Holotype (Fig. 11a). Length 2.80 mm. Color black; antenna and tarsi ferruginous. Body with marked constriction between pronotum and elytron; in profile convex. Rostrum with median costa and pair of submedian costae; intervening furrows with rows of coarse punctures each containing one mesad directed seta; in apical third punctate; base dorsally protruding, projecting from profile subangularly. Forehead coarsely punctate. Pronotum with sides weakly converging, rounded to distinct subapical constriction; disk coarsely punctate; along midline with row of ca. 16 punctures; interspaces microreticulate; with median costa. Elytra cuneiform; from humeri markedly converging to narrow apex; dorsally somewhat flattened; base bisinuate; striae deeply incised; intervals costate, with 1-2 rows of small punctures. Legs. Femora densely punctate. Profemur with subbasal callus anteriorly projecting. Tibiae subbasally with dorsal angulation; metatibia subapically with suprauncal denticle. Abdominal ventrites 1-2 laterally swollen, medially forming common cavity; ventrite 5 coarsely punctate, weakly concave. Penis (Fig. 10b) with sides of body diverging; apex subtruncate, with short median extension; endophallus with pair of elongate sclerites, from ostium almost reaching asymmetrical bell-shaped transfer apparatus; ductus ejaculatorius without bulbus. **Intraspecific variation.** Length 2.42–3.05 mm. Body of females subovate. Female rostrum dorsally somewhat flattened; median costa and pair of submedian costae subglabrous.

##### Material examined.

Holotype (ANIC): ARC4047 (PCR failed), Queensland, Daintree N.P., Noah Beach, S16°09', E145°26', FIT N03F, 10 m, 15-III-07-V-1998. Paratypes (ANIC, SMNK): Queensland: 2 exx, same data as holotype; 1 ex, Noah Beach, S16°09.07', E145°26.45', FIT=4, 10 m, 07-I-09-II-1998; 4 exx, ARC4048 (PCR failed), Noah Beach, S16°09', E145°26', FIT N04F, 10 m, 09-II-15-III-1998.

##### Distribution.

Queensland: Daintree National Park.

##### Biology.

Sifted from leaf litter in primary forest.

##### Etymology.

This epithet is an adjective based on the name of the type locality, the Daintree National Park.

#### 12. Trigonopterus deplanatus

Taxon classificationAnimaliaColeopteraCurculionidae

Riedel sp. n.

http://zoobank.org/A0B05E4E-B897-4A78-941D-E0B9F4B48EB3

##### Diagnostic description.

**Figure 12.**
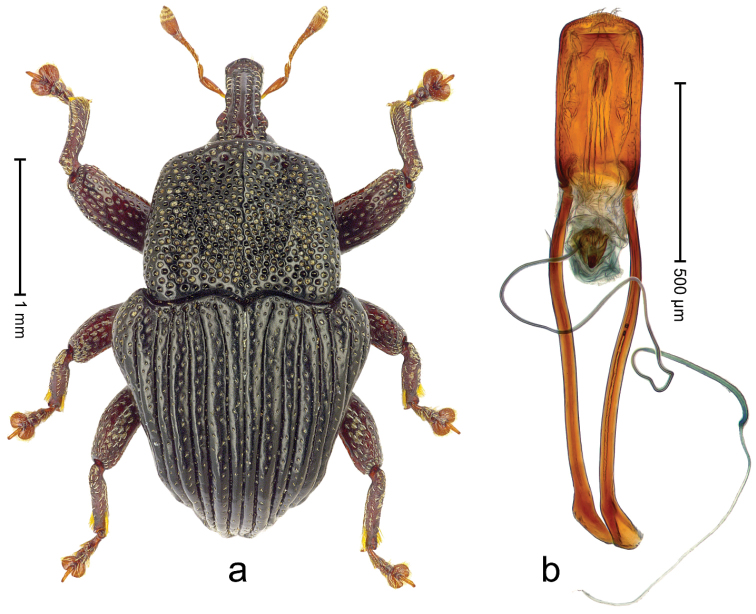
*Trigonopterus deplanatus* sp. n., holotype; **a** Habitus **b** Penis.

Holotype (Fig. 12a). Length 2.98 mm. Color black; antenna and legs ferruginous. Body with marked constriction between pronotum and elytron; in profile convex. Rostrum in basal half with median costa and pair of submedian costae, intervening furrows with rows of mesad directed setae; in apical third scabrous; epistome posteriorly with transverse ridge; base dorsally weakly protruding, weakly projecting from profile. Forehead coarsely punctate. Pronotum subquadrate, sides weakly converging, apex subtruncate; disk punctate-reticulate; interspaces between punctures narrow, partly broken away; along midline with row of ca. 20-23 punctures; in anterior half with median ridge bordered by pair of shallow depressions. Elytra cuneiform; from humeri markedly converging to narrow apex; dorsally flattened; base bisinuate; striae deeply incised; intervals costate, punctate with small punctures. Legs. Femora densely punctate. Profemur with subbasal callus anteriorly projecting. Tibiae subbasally with dorsal angulation. Abdominal ventrites 1 laterally swollen, medially concave, with course punctures; abdominal ventrite 2 swollen; ventrite 5 densely punctate, weakly concave. Penis (Fig. 12b) with sides of body subparallel; apex subangulate; transfer apparatus funnel-shaped, with asymmetrical extension on one side; ductus ejaculatorius without bulbus.

##### Material examined.

Holotype (QMBA): ARC3893 (PCR failed), Queensland, Cairns, Mt. Williams, S16°55', E145°40', 850 m, rainforest, sieved litter, Berlesate 868, 03-XII-1993.

##### Distribution.

Queensland: Mt. Williams.

##### Biology.

Sifted from leaf litter in primary forest.

##### Etymology.

This epithet is based on the Latin participle *deplanatus* (levelled) and refers to the dorsally flattened body.

#### 13. Trigonopterus evanidus

Taxon classificationAnimaliaColeopteraCurculionidae

(Pascoe)

Idotasia evanida Pascoe, 1872: 100.
Trigonopterus evanidus (Pascoe): Pullen et al. 2014: 271.

##### Diagnostic description.

**Figure 13.**
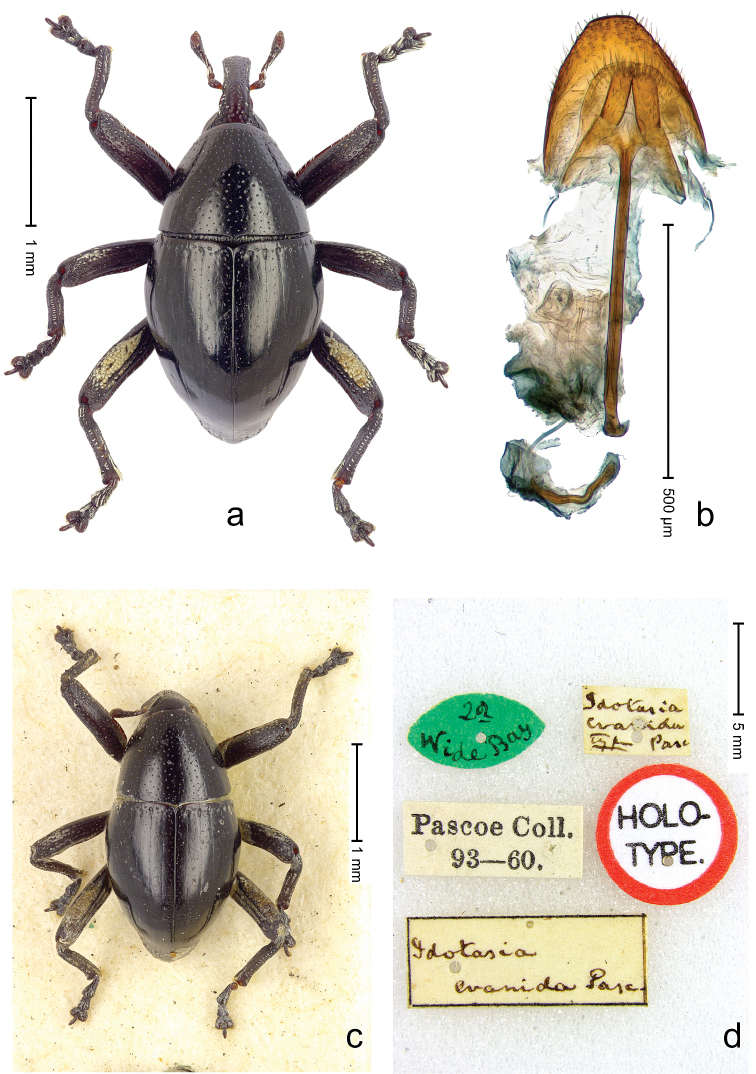

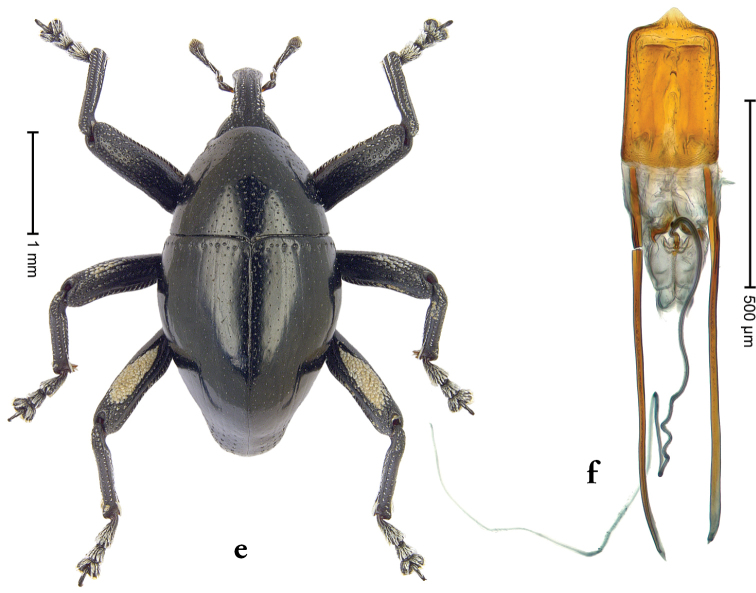
*Trigonopterus evanidus* (Pascoe), female lectotype; **a** Habitus **b** Terminalia **c** as mounted originally **d** original labels. *Trigonopterus evanidus* (Pascoe), male; **e** Habitus **f** Penis.

Male (ARC3662; Fig. 13e). Length 3.19 mm. Color black. Body subovate, almost without constriction between pronotum and elytron; in profile evenly convex. Rostrum with median costa and pair of submedian ridges; intervening furrows with rows of white scales; apical 1/3 rugose-punctate. Eyes with dorsal margin bordered by furrow. Forehead sparsely punctate. Pronotum with disk densely punctate with small punctures; interspaces not microreticulate; sides foveate; base medially weakly extended towards elytral suture. Elytra subglabrous, striae marked by very shallow lines; along base and humeri with row of large punctures; apex with dense rows of small shallow punctures. Legs. Femora microreticulate, punctate. Metafemur dorsally with elongate patch of dense white scales; posterior surface with ventral edge rimmed by costa and row of scales, with longitudinal furrow containing row of scales parallel to indistinct dorsoposterior edge. Mesotibia apically with uncus and larger premucro fused in basal half, diverging in apical half. Metatibia apically with uncus and small angular premucro. Abdominal ventrite 2 swollen, with posterior edge projecting, medially forming common cavity with ventrite 1; ventrite 5 concave, dull, microreticulate, punctate. Penis (Fig. 13f) with sides of body subparallel; apex with median triangular extension somewhat confluent with outline of apex; transfer apparatus short, dentiform, bordered by S-shaped sclerites; ductus ejaculatorius without bulbus. **Female lectotype** (Fig. 13a–d). As male except: length 2.56 mm. Rostrum punctate-rugose, with weak median costa. Mesotibia apically with large uncus and much smaller premucro. Terminalia (Fig. 12b) with styli wide.

##### Material examined.

Type specimens. Female, lectotype by present designation (BMNH): Queensland, Wide Bay (labels Fig. 12d), ARC4080 (PCR failed). Other specimens (QMBA, SMNK): Queensland: 1 ex, ARC3660 (EMBL # LN888164), Brisbane, St. Lucia, S27°30.033', E152°59.562', 27 m, hand-collected from *Mallotus* leaves, 20-XI-2013; 2 exx, ARC3661 (EMBL # LN888165), ARC3662 (EMBL # LN888166), Brisbane, St. Lucia, S27°30.033', E152°59.562', 27 m, hand-collected from *Mallotus* leaves, 23-XI-2013; 1 ex, ARC3862 (EMBL # LN888246), Ventnor Site 1, FIT trap, S24°53.58', E151°19.98', 475 m, 01-X-05-XII-2013.

##### Distribution.

Queensland: Brisbane.

##### Biology.

Collected from foliage in gardens and forests.

##### Notes.

The lectotype here designated has a circular label reading “Holotype” fixed to its pin by staff of the BMNH, but Pascoe (1872) did not designate a holotype in the original description nor specify the number of specimens examined. As other syntypes may exist, we here designate the one in the BMNH as the lectotype to ensure stability of nomenclature in case additional syntypes are discovered that belong to different species.

#### 14. Trigonopterus finniganensis

Taxon classificationAnimaliaColeopteraCurculionidae

Riedel sp. n.

http://zoobank.org/52761014-3B8F-4562-9DE2-5A14026E5A62

##### Diagnostic description.

**Figure 14.**
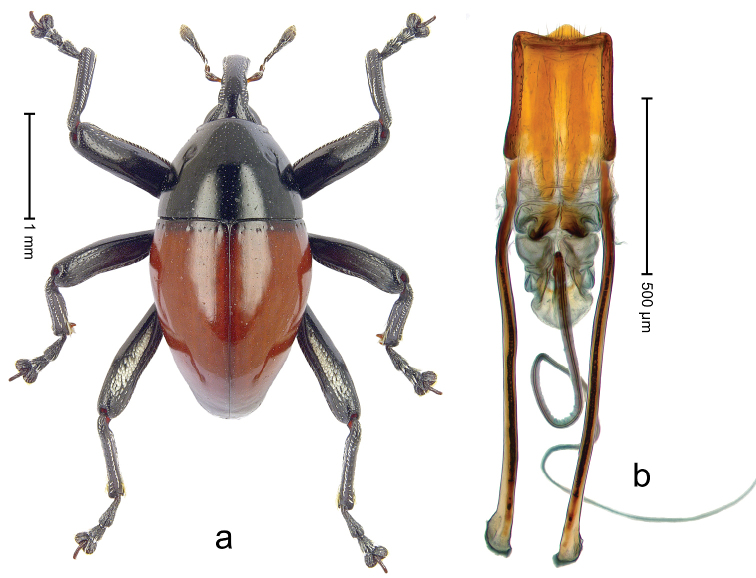
*Trigonopterus finniganensis* sp. n., holotype; **a** Habitus **b** Penis.

Holotype (Fig. 14a). Length 2.98 mm. Color black, elytra orange-ferruginous. Body subovate, almost without constriction between pronotum and elytron; in profile evenly convex. Rostrum dorsally sparsely punctate, with pair of shallow sublateral furrows containing sparse rows of mesad-directed scales. Eyes with dorsal margin weakly carinate, bordered by furrow. Forehead with sparse minute punctures. Pronotum with disk subglabrous, with minute punctures; sides above coxa with scattered coarse punctures; base medially weakly extended towards elytral suture. Elytra subglabrous; along base and humeri with sparse row of large, shallow punctures; apex with scattered shallow punctures. Legs. Femora weakly microreticulate, with small punctures. Metafemur dorsally with elongate patch of dense silvery scales; posterior surface with pair of longitudinal furrows containing rows of indistinct scales parallel to ventral and dorsal edge; dorsoposterior edge indistinct. Mesotibia apically with uncus and larger premucro approximate at base, not fused, widely diverging. Metatibia apically with uncus and angular premucro. Abdominal ventrite 2 swollen, with posterior edge projecting, medially forming common cavity with ventrite 1; ventrite 5 weakly concave, subglabrous, dull, with sparse minute punctures. Penis (Fig. 14b) with sides of body subparallel, weakly concave; apex with median triangular extension confluent with outline of apex; transfer apparatus short, spiniform, apically bordered by pair of P-shaped sclerites; ductus ejaculatorius without bulbus. **Intraspecific variation.** Length 2.46–2.98 mm. Female rostrum dorsally subglabrous, densely punctate with small punctures. Mesotibia apically with large uncus and much smaller premucro. Female abdominal ventrites 1 and 2 medially flat; female abdominal ventrite 5 flat.

##### Material examined.

Holotype (QMBA): ARC3702 (EMBL # LN888184), Queensland, Mt. Finnigan, ascent from Shiptons Flat, S15°49.043', E145°16.780', 1055 m, 28-IV-2014. Paratypes (QMBA, SMNK): 3 exx, ARC3703 (EMBL # LN888185), ARC3704 (EMBL # LN888186), ARC3705 (EMBL # LN888187), same data as holotype; 2 exx, 3.5 km NNE Mt. Spurgeon, 16°24', S 145°13', E, 16-X-1991, Pyrethrum, trees & rocks; 2 exx, Mt. Finnigan Summit, via Helenvale, 03-05-XII-1990, 1050 m.

##### Distribution.

Queensland (Mt. Finnigan, Mt. Spurgeon).

##### Biology.

Beaten from foliage of montane sclerophyll shrubland.

##### Etymology.

This epithet is an adjective based on the name of the type locality, Mt. Finnigan.

##### Notes.

*Trigonopterus finniganensis* Riedel, sp. n. was coded as “*Trigonopterus* sp. 565”.

#### 15. Trigonopterus fraterculus

Taxon classificationAnimaliaColeopteraCurculionidae

Riedel sp. n.

http://zoobank.org/BAA81DE2-8A8D-438B-8CBF-6FA42DEC5513

##### Diagnostic description.

**Figure 15.**
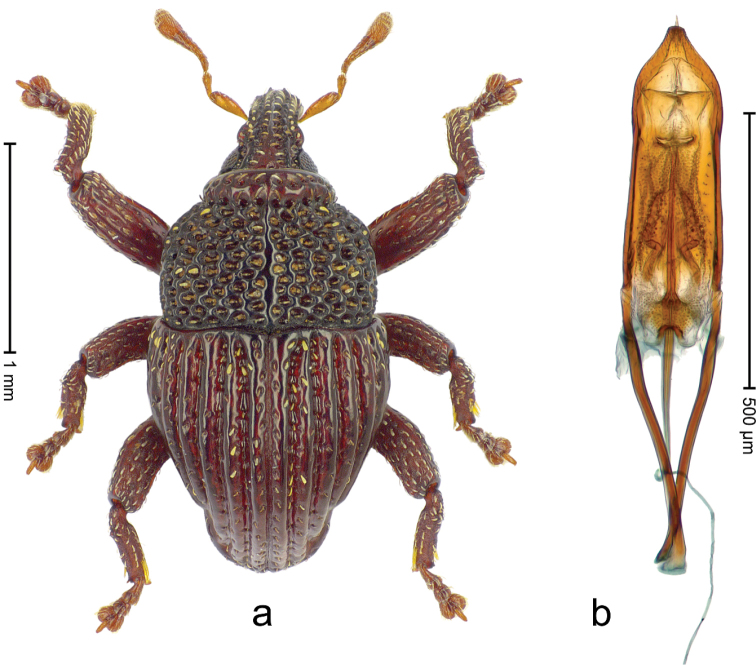
*Trigonopterus fraterculus* sp. n., holotype; **a** Habitus **b** Penis.

Holotype (Fig. 15a). Length 1.92 mm. Color ferruginous; pronotum dark ferruginous, almost black. Body subovate, with marked constriction between pronotum and elytron; in profile weakly convex. Rostrum with median ridge and pair of submedian ridges; intervening furrows with rows of coarse punctures each containing one mesad directed scale; epistome posteriorly with angulate ridge bearing 4 denticles. Forehead coarsely punctate-rugose. Pronotum with distinct subapical constriction; disk foveate-reticulate; each fovea containing one brown scales; sublaterally few scales widened and cream-colored; with irregular median costa. Elytra with striae deeply incised; intervals costate, subglabrous, with sparse recumbent scales; few scales almond-shaped, cream-colored; base weakly bisinuate. Legs. Femora punctate-rugose, with sparse recumbent scales. Tibiae subbasally with dorsal angulation; metatibia subapically with suprauncal denticle. Abdominal venter with coarse punctures containing upcurved clavate scales; ventrite 5 basally with transverse ridge. Penis (Fig. 15b) with sides of body weakly converging, pointed apex extended, curved ventrad; endophallus denticulate, with pair of lyriform sclerites; transfer apparatus long, spiniform; ductus ejaculatorius near insertion to transfer apparatus sclerotized, without bulbus. **Intraspecific variation.** Length 1.92–2.20 mm. Female body slender. Female rostrum dorsally somewhat flattened; with median costa and pair of submedian costae; epistome simple.

##### Material examined.

Holotype (ANIC): ARC4043 (PCR failed), Queensland, 11 km ENE of Mt. Tozer, S12°43', E143°18', rainforest litter, Berlesate ANIC 1062, 11-16-VII-1986. Paratypes (ANIC, SMNK): Queensland: 2 exx, same data as holotype; 1 ex, 9 km ENE of Mt. Tozer, S12°43', E143°17', open forest litter, Berlesate ANIC 1061, 05-10-VII-1986.

##### Distribution.

Queensland: Iron Range.

##### Biology.

Sifted from leaf litter in primary forest.

##### Etymology.

This epithet is based on the Latin noun *fraterculus* (younger brother) and refers to its presumably close phylogenetic relationship to the larger species *Trigonopterus australis* Riedel, sp. n..

#### 16. Trigonopterus garradungensis

Taxon classificationAnimaliaColeopteraCurculionidae

Riedel sp. n.

http://zoobank.org/8BC90C74-FC26-42F7-A7C6-88A7F7EA4ADC

##### Diagnostic description.

**Figure 16.**
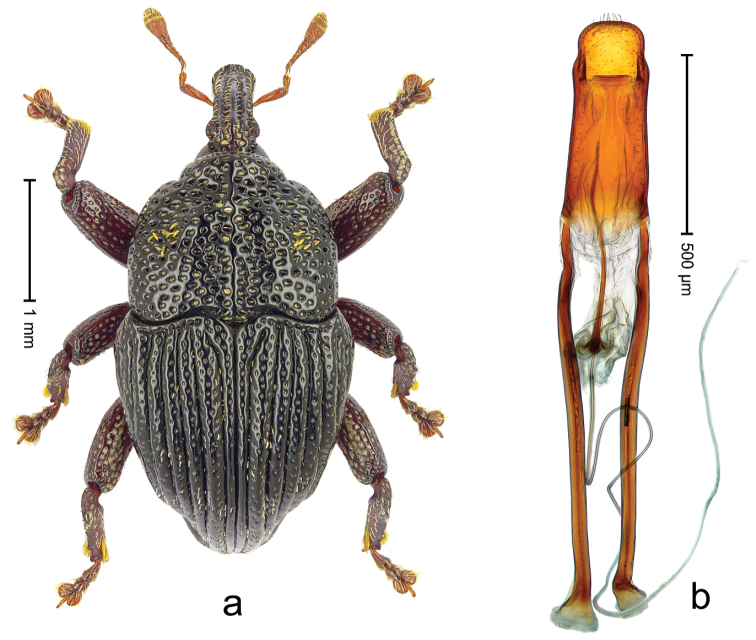
*Trigonopterus garradungensis* sp. n., holotype; **a** Habitus **b** Penis.

Holotype (Fig. 16a). Length 3.28 mm. Color black; antenna and legs ferruginous. Body subovate, with marked constriction between pronotum and elytron; in profile convex. Rostrum with median ridge and pair of submedian ridges ending in apical third; intervening furrows with rows of coarse punctures each containing one mesad directed seta; base dorsally protruding, markedly projecting from profile subangularly; epistome posteriorly with angulate ridge bearing 4 denticles. Forehead coarsely punctate-rugose. Pronotum with subapical constriction; disk coarsely punctate-reticulate; with median costa; near middle with pair of weak swellings, further laterad with clusters of sparse yellow recumbent scales. Elytra with striae deeply incised; intervals costate, punctate, with few scattered recumbent scales; sutural interval narrow, below level of interval 2; base markedly bisinuate. Legs. Femora densely punctate. Profemur with subbasal callus anteriorly projecting. Tibiae subbasally with dorsal angulation; metatibia in apical third with blunt suprauncal projection. Abdominal ventrites 1-2 laterally swollen, medially forming common cavity; ventrite 5 punctate, weakly concave. Penis (Fig. 16b) with sides of body weakly converging, in apical third with constriction, apex rounded; transfer apparatus flagelliform, ca. 1.2× longer than body of penis; ductus ejaculatorius without bulbus. **Intraspecific variation.** Length 2.97–3.28 mm. Female rostrum dorsally somewhat flattened; median costa and pair of submedian costae subglabrous; epistome simple. Female abdominal ventrites 1-2 medially flat; ventrite 5 coarsely punctate, basally swollen, apically flat.

##### Material examined.

Holotype (QMBA): ARC3732 (EMBL # LN888214), Queensland, Garradunga, Polly Ck., N Innisfail, Hasenpusch property, S17°27.458', E146°01.227', sample 4, 82 m, 16-IV-2014. Paratypes (QMBA, SMNK): Queensland: 3 exx, ARC3733 (EMBL # LN888215), ARC3734 (EMBL # LN888216), ARC3735 (EMBL # LN888217), same data as holotype; 2 exx, Garradunga, Polly Ck., N Innisfail, Hasenpusch property, S17°27.388', E146°01.200', sample 1, 70 m, 16-IV-2014; 3 exx, Garradunga, Polly Ck., N Innisfail, Hasenpusch property, S17°27.306', E146°01.214', sample 2, 103 m, 16-IV-2014; 1 ex, Garradunga, Polly Ck., N Innisfail, Hasenpusch property, S17°27.458', E146°01.227', sample 4, 82 m, 16-IV-2014.

##### Distribution.

Queensland: Garradunga.

##### Biology.

Sifted from leaf litter in primary forest.

##### Etymology.

This epithet is an adjective based on the name of the type locality, Garradunga.

##### Notes.

*Trigonopterus garradungensis* Riedel, sp. n. was coded as “*Trigonopterus* sp. 559”.

#### 17. Trigonopterus hasenpuschi

Taxon classificationAnimaliaColeopteraCurculionidae

Riedel sp. n.

http://zoobank.org/63796EB2-1187-4C40-8B57-75563B3BA9B8

##### Diagnostic description.

**Figure 17.**
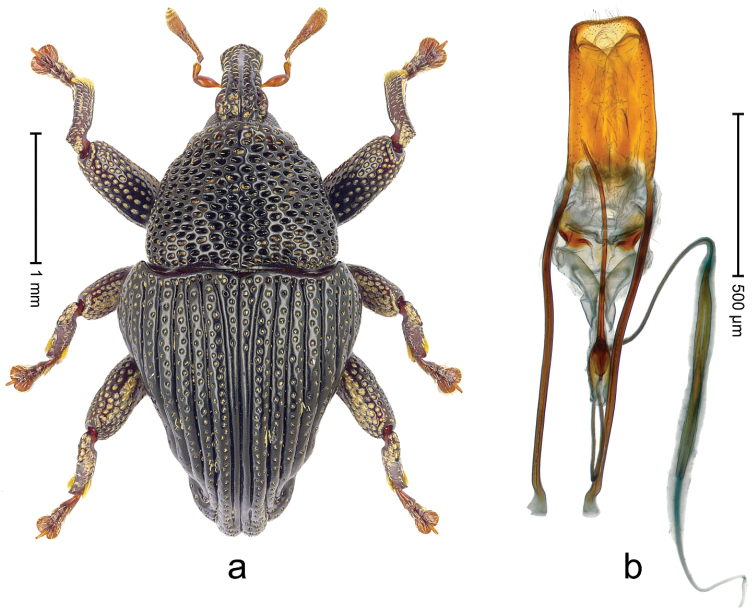
*Trigonopterus hasenpuschi* sp. n., holotype; **a** Habitus **b** Penis.

Holotype (Fig. 17a). Length 3.22 mm. Color black; antenna and tarsi ferruginous. Body subrhomboid, with marked constriction between pronotum and elytron; in profile convex. Rostrum with median ridge and pair of submedian ridges; intervening furrows with rows of coarse punctures each containing one mesad directed seta; base dorsally protruding, markedly projecting from profile subangularly; epistome posteriorly with subangulate ridge. Forehead coarsely punctate-rugose. Pronotum with sides converging to apex, almost without subapical constriction; foveate-reticulate; each fovea containing one inconspicuous seta; interspaces subglabrous. Elytra cuneiform, from broad rounded humeri markedly converging to narrow apex; base bisinuate; striae deeply incised; intervals costate, with 1-2 rows of small punctures; sutural interval subglabrous except few punctures near base; intervals 2-5 behind middle with inconspicuous transverse patches of narrow recumbent scales. Legs. Femora densely punctate. Profemur with subbasal callus anteriorly projecting. Tibiae subbasally with dorsal angulation; pro- and metatibia subapically with suprauncal denticle. Abdominal ventrites 1-2 laterally swollen, medially forming common cavity; ventrite 5 coarsely punctate, weakly concave. Penis (Fig. 17b) with sides of body subparallel; apex subtruncate; endophallus with pair of subtriangular sclerites; transfer apparatus flagelliform; ductus ejaculatorius with distinct bulbus. **Intraspecific variation.** Length 2.59–3.22 mm. Body in smaller specimens and females more slender. Female rostrum dorsally somewhat flattened; median ridge and pair of submedian costae subglabrous; epistome simple. Female abdominal ventrite 5 flat.

##### Material examined.

Holotype (QMBA): ARC3723 (EMBL # LN888205), Queensland, Mission Beach, Clump Mt. N.P., Bicton Hill, S17°50.499', E146°05.905', sample 1, 150 m, 15-IV-2014. Paratypes (QMBA, SMNK): Queensland: 3 exx, ARC3724 (EMBL # LN888206), ARC3725 (EMBL # LN888207), ARC3726 (EMBL # LN888208), same data as holotype; 2 exx, ARC3740 (EMBL # LN888222), ARC3741 (EMBL # LN888223), Garradunga, Polly Ck., N Innisfail, Hasenpusch property, S17°27.388', E146°01.200', sample 1, 70 m, 16-IV-2014; 1 ex, Garradunga, Polly Ck., N Innisfail, Hasenpusch property, S17°27.306', E146°01.214', sample 2, 103 m, 16-IV-2014; 2 exx, Garradunga, Polly Ck., N Innisfail, Hasenpusch property, S17°27.252', E146°01.222', sample 3, 105 m, 16-IV-2014; 1 ex, ARC3731 (EMBL # LN888213), Garradunga, Polly Ck., N Innisfail, Hasenpusch property, S17°27.458', E146°01.227', sample 4, 82 m, 16-IV-2014; 3 exx, Stone Ck. (Hasenpusch), 01-XI-1995-06-II-1996, 100 m, pitfall traps, S17°28', E146°01'; 2 exx, Kirrama Range, 09-XII-1986, Berlesate 730, S18°10', E145°45', rainforest, 700 m, sieved litter; 1 ex, Cardwell range, Upper Broadwater Ck. Valley, 18-XII-1986-14-I-1987, 750 m, RF, Pitfall Traps.

##### Distribution.

Queensland: Mission Beach, Cardwell Range, Kirrama Range, Garradunga.

##### Biology.

Sifted from leaf litter in primary forest.

##### Etymology.

This species is named in honor of Jack Hasenpusch (Garradunga), who preserves the habitat of this and other *Trigonopterus* species on his insect farm.

##### Notes.

*Trigonopterus hasenpuschi* Riedel, sp. n. was coded as “*Trigonopterus* sp. 554”.

#### 18. Trigonopterus hartleyensis

Taxon classificationAnimaliaColeopteraCurculionidae

Riedel sp. n.

http://zoobank.org/49E719CC-0A8C-4C07-AAE1-9BB31D846F98

##### Diagnostic description.

**Figure 18.**
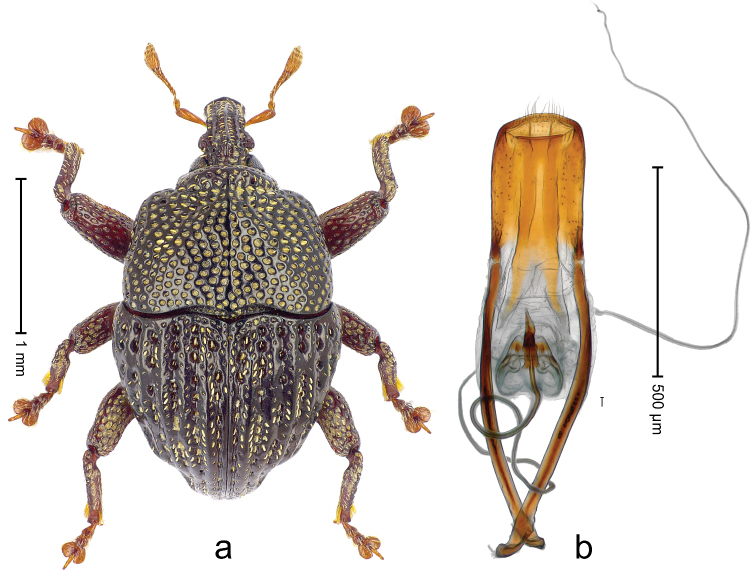
*Trigonopterus hartleyensis* sp. n., holotype; **a** Habitus **b** Penis.

Holotype (Fig. 18a). Length 2.14 mm. Color black; antenna and legs ferruginous. Body subglobose, with shallow constriction between pronotum and elytron; in profile convex. Rostrum with median ridge and pair of submedian ridges; intervening furrows with rows of mesad directed setae; base dorsally protruding, markedly projecting from profile; epistome posteriorly with transverse ridge. Forehead coarsely punctate-rugose. Pronotum broad, with sides weakly converging to apex, with distinct subapical constriction; punctate-reticulate; with sparse, narrow, cream-colored scales; in anterior half with indistinct median ridge. Elytra with striae marked by isolated foveae; intervals costate; with sparse, recumbent, cream-colored scales; base bisinuate. Legs. Femora densely punctate. Tibiae subbasally with dorsal angulation. Abdominal ventrites 1-2 foveate; ventrite 1 concave; ventrite 2 swollen, transversely costate; ventrite 5 subbasally with shallow impression. Penis (Fig. 18b) with sides of body subparallel; apex rounded; endophallus with pair of elongate sclerites; transfer apparatus short, spiniform; ductus ejaculatorius without bulbus. **Intraspecific variation.** Length 1.90–2.40 mm. Female body subovate. Female rostrum dorsally somewhat flattened; in apical half with submedian rows of punctures; near base with median costa and pair of submedian costae; epistome simple.

##### Material examined.

Holotype (QMBA): ARC3762 (EMBL # LN888239), Queensland, Cedar Bay N.P., road between Rossville and Bloomfield, S15°47.510', E145°18.141', sample 2-B, 322 m, 01-V-2014. Paratypes (ANIC, QMBA, SMNK): Queensland: 2 exx, ARC3763 (EMBL # LN888240), same data as holotype; 11 exx, ARC3764 (EMBL # LN888241), ARC3765 (EMBL # LN888242), ARC3766 (EMBL # LN888243), 2.5 km W Mt. Hartley, near Rossville – Bloomfield-road, S15°47.071', E145°18.701', sample 1, 649 m, 01-V-2014; 1 ex, Cedar Bay N.P., road between Rossville and Bloomfield, S15°47.510', E145°18.141', sample 2, 322 m, 29-IV-2014; 5 exx, 2.5 km W Mt. Hartley, near Rossville – Bloomfield-road, S15°47.393', E145°18.348', sample 3, 419 m, 01-V-2014; 1 ex, Big Tableland, 740 m, 20-XII-1990-08-I-1991, flight intercept trap, S15°43', E145°17'; 1 ex, 2.5 km S Mt. Hartley, 08-XII-1993-02-II-1994, pitfalls, S15°47', E145°19'; 7 exx, Mt Hartley, 30 km S Cooktown, 760 m, SBP62, rainforest litter, 03-VII-1982; 4 exx, Mt. Finnigan, 30 km S Cooktown, 400 m, litter and fungi, rainforest, 01-VII-1982; 2 exx, Mt. Finnigan, 30 km S Cooktown, 400 m, moist litter pockets rainforest, 03-VII-1982; 1 ex, Moses Ck, 4 km NbyE of Mt. Finnigan, Berlesate ANIC 696, sieved rainforest litter, 14-16-X-1980.

##### Distribution.

Queensland: Surroundings of Mt. Hartley.

##### Biology.

Sifted from leaf litter in primary forest.

##### Etymology.

This epithet is an adjective based on the name of the type locality, Mt. Hartley.

##### Notes.

*Trigonopterus hartleyensis* Riedel, sp. n. was coded as “*Trigonopterus* sp. 555”.

#### 19. Trigonopterus kurandensis

Taxon classificationAnimaliaColeopteraCurculionidae

Riedel sp. n.

http://zoobank.org/693B815E-07A2-4AC3-A8C4-7F2C2030D0E8

##### Diagnostic description.

**Figure 19.**
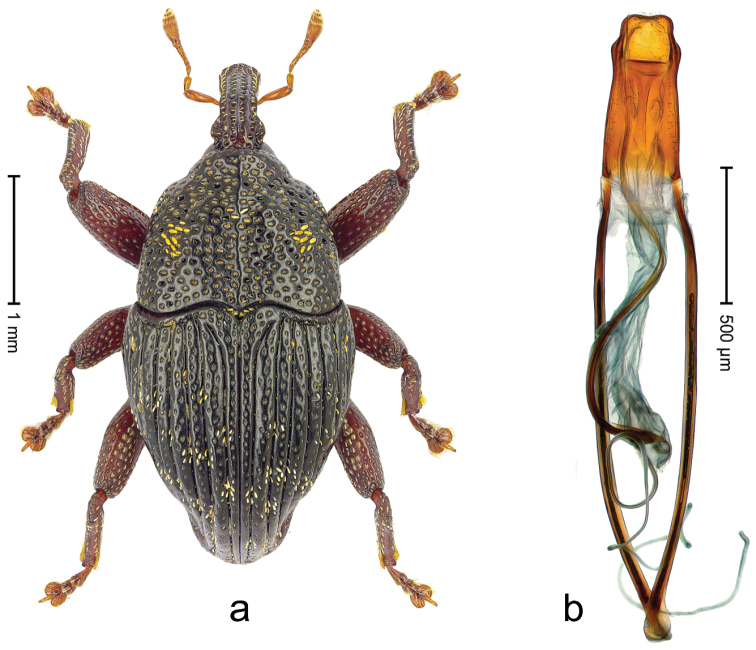
*Trigonopterus kurandensis* sp. n., holotype; **a** Habitus **b** Penis.

Holotype (Fig. 19a). Length 3.19 mm. Color black; antenna and legs ferruginous. Body subovate, with shallow constriction between pronotum and elytron; in profile convex. Rostrum with median ridge and pair of submedian ridges ending in apical third; intervening furrows with rows of coarse punctures each containing one mesad directed seta; base dorsally protruding, markedly projecting from profile subangularly; epistome posteriorly with curved ridge bearing 4 denticles. Forehead coarsely punctate-rugose. Pronotum with subapical constriction; disk coarsely punctate-reticulate; with median costa; near middle with pair of weak swellings, further laterad with clusters of sparse yellow recumbent scales. Elytra with striae deeply incised; intervals costate, microreticulate, punctate, with few scattered recumbent scales; base markedly bisinuate. Legs. Femora densely punctate. Profemur with subbasal callus anteriorly projecting. Tibiae subbasally with dorsal angulation; metatibia in apical third with blunt suprauncal projection. Abdominal ventrites 1-2 laterally swollen, medially forming common cavity; ventrite 5 punctate, weakly concave. Penis (Fig. 19b) with sides of body weakly converging, in apical third with constriction, apex rounded; transfer apparatus flagelliform, ca. 3.0× longer than body of penis; ductus ejaculatorius without bulbus. **Intraspecific variation.** Length 2.97–3.19 mm. Female body more slender, surface more polished. Female rostrum dorsally somewhat flattened; median costa and pair of submedian costae subglabrous; epistome simple. Female abdominal ventrites 1-2 medially flat; ventrite 5 coarsely punctate, basally swollen, apically flat.

##### Material examined.

Holotype (QMBA): ARC3711 (EMBL # LN888193), Queensland, Kuranda N.P., Saddle Mountain Road, S16°49.094', E145°39.712', sample 2, 637 m, 31-III-2014. Paratypes (ANIC, QMBA, SMNK): Queensland: 10 exx, ARC3710 (EMBL # LN888192), ARC3712 (EMBL # LN888194), ARC3713 (EMBL # LN888195), same data as holotype; 2 exx, Kuranda N.P., Saddle Mountain Road, S16°49.106', E145°39.759', sample 1, 637 m, 31-III-2014; 1 ex, ARC4052 (PCR failed), Kuranda, Black Mt. Rd., S17°47', E145°39', rainforest, sieved litter, Q.M. Berlesate No. 223, 360 m, 09-VI-1980; 4 km W of Kuranda, S16°49', E145°36', ANIC Berlesate 340, 450 m, 27-VI-1971.

##### Distribution.

Queensland: Kuranda.

##### Biology.

Sifted from leaf litter in primary forest.

##### Etymology.

This epithet is an adjective based on the name of Kuranda, the type locality.

##### Notes.

*Trigonopterus kurandensis* Riedel, sp. n. was coded as “*Trigonopterus* sp. 558”.

#### 20. Trigonopterus laetus

Taxon classificationAnimaliaColeopteraCurculionidae

(Lea)

Idotasia laeta Lea, 1913: 610.
Trigonopterus laetus (Lea): Pullen et al. 2014: 271.

##### Diagnostic description.

**Figure 20.**
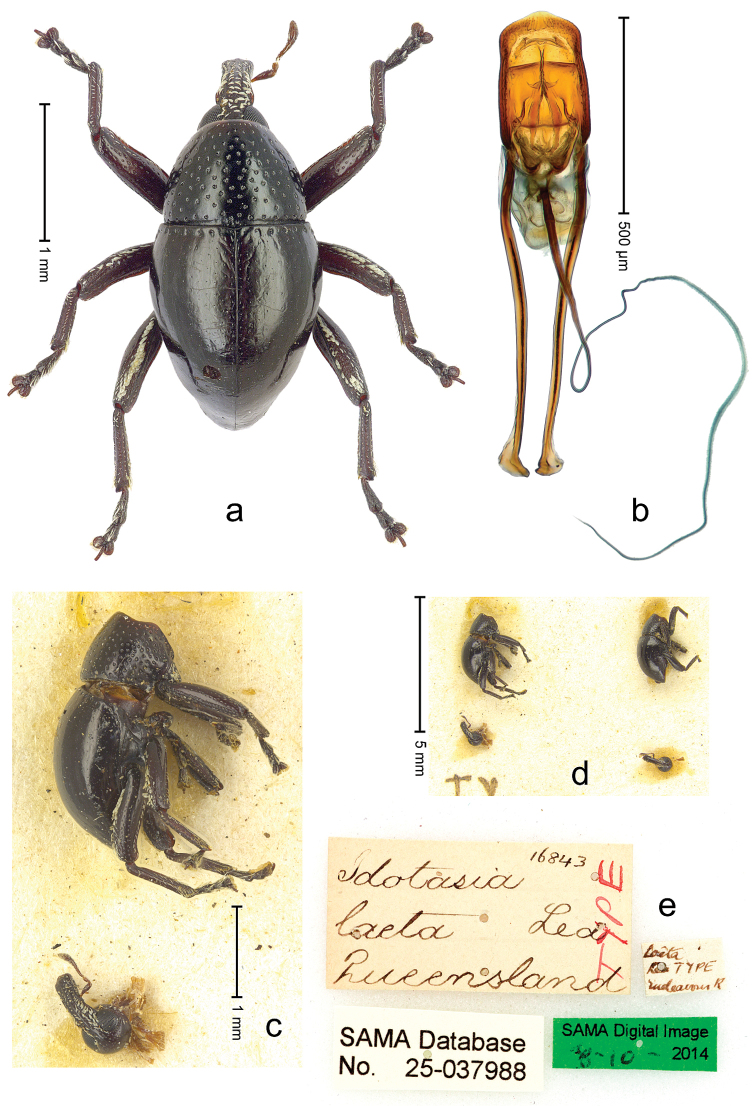
*Trigonopterus laetus* (Lea), male lectotype; **a** Habitus **b** Penis **c** as mounted originally **d** original labels.

Lectotype (Fig. 20a, c). Length 2.30 mm. Color black, legs and antenna dark ferruginous. Body subovate, with constriction between pronotum and elytron; in profile with shallow constriction. Rostrum with median ridge and pair of submedian ridges; intervening furrows with rows of white scales; apical 1/3 rugose-punctate. Eyes large, in dorsal position. Pronotum with disk punctate; sides more densely punctate with slightly larger punctures. Elytra subglabrous; along base and humeri with row of large punctures; subapically with sparse small punctures. Legs. Meso- and metafemur dorsally with narrow band of white scales; anteroventral ridge of femora weakly crenulate, terminating 1/3 before apex with minute denticle. Tibial apex with uncus, without premucro. Abdominal ventrite 2 posteriorly forming edge; medially forming common cavity with ventrite 1; ventrite 5 weakly concave, almost flat, nude, microreticulate, sparsely punctate. Penis (Fig. 20b) with sides of body subparallel; apex broadly subangulate; body containing two pairs of lyriform sclerites; transfer apparatus short, dentiform; ductus ejaculatorius near insertion to transfer apparatus swollen, subapically with very indistinct bulbus. **Intraspecific variation.** Length 1.96–2.30 mm. Female rostrum subglabrous, with submedian rows of punctures. Female abdominal ventrites 1 and 2 convex, medially flat.

##### Material examined.

Type specimens. Male, lectotype by present designation (Fig. 20) (SAMA): Queensland, Endeavour River (labels Fig. 20e), ARC4038 (PCR failed). Female, paralectotype (SAMA), same data as lectotype; 1 paralectotype (ANIC – permanent loan from Macleay Museum), Sue Island. Other specimens (ANIC, QMBA, SMNK): Queensland: 1 ex, 44 km N of Cairns, beating shrubs, 10-XII-1982; 3 exx, ARC1672 (EMBL # LN888161), ARC1673 (EMBL # LN888162), ARC1674 (EMBL # LN888163), Cooktown, Jensen´s Xing, Pyrethrum, trees & logs, RF, S15°26', E145°07', 20 m, 19-22-X-2008; 1 ex, ARC3692 (EMBL # LN888179), Cooktown, Mt. Cook N.P., S15°28.648', E145°15.793', to S15°29.252', E145°15.992', 63-324 m, 24-IV-2014; 2 exx, Cooktown, Mt. Cook N.P., S15°29', E145°16',11-12-X-1980; 1 ex, Mt. Webb N.P., S15°04', E145°07', ex malaise trap, 27-30-IV-1981; 1 ex, Mt. Webb N.P., S15°04', E145°07', 28-30-IX-1980; 2 exx, Mt. Webb N.P., S15°04', E145°07', 30-IX-1980; 3 exx, Mt. Webb N.P., S15°04', E145°07', 27-30-IV-1981; 3 exx, Mt. Webb N.P., S15°04', E145°07', 29-IX-1980; 3 exx, 11 km ENE of Mt. Tozer, S12°43', E143°18', beating rainforest vegetation, 11-16-VII-1986; 9 exx, 9 km ENE of Mt. Tozer, S12°43', E143°17', beating rainforest vegetation, 05-10-VII-1986; 4 exx, 3 km ENE of Mt. Tozer, S12°44', E143°14', beating, 11-16-VII-1986; 1 ex, 3 km ENE of Mt. Tozer, S12°44', E143°14', 01-04-VII-1986; 1 ex, 8 km E by N of Mt. Tozer, S12°44', E143°17', beating rainforest vegetation, 07-VII-1986; 1 ex, 15 km NE by E Heathlands, sweeping, S11°41', E142°42', 15-26-I-1992; 1 ex: West Claudie River, 4 km SW road junction, 12°44', S 143°15', E, 11-XII-1986, malaise; 1 ex, 3 km E Lockerbie, Cape York, Pyrethrum on logs, Rf, 19-23-III-1987; 2 exx, Bamaga, XII-1983.

##### Distribution.

Queensland: Cooktown, Mt. Webb N.P., Heathlands N.P., Iron Range N.P., Lockerbie Scrub.

##### Biology.

Beaten from foliage in rainforest.

##### Notes.

Lea (1913) did not designate a holotype in the original description nor specify the number of specimens examined. The original description is based on more than one specimen. One pair with the male marked “TY” and one syntype from Sue Island could be examined, but other specimens may exist. The male is here designated as lectotype.

#### 21. Trigonopterus lewisensis

Taxon classificationAnimaliaColeopteraCurculionidae

Riedel sp. n.

http://zoobank.org/252B8FC8-CC37-4D95-9B9B-B38366B15055

##### Diagnostic description.

**Figure 21.**
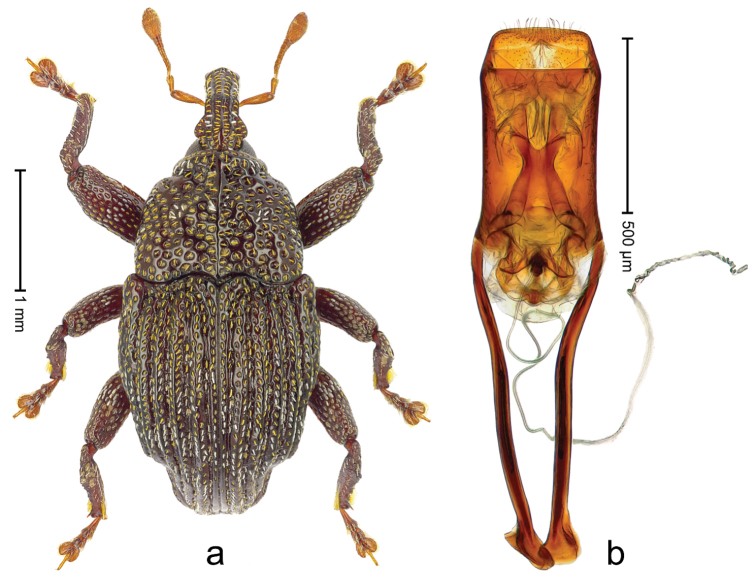
*Trigonopterus lewisensis* sp. n., holotype; **a** Habitus **b** Penis.

Holotype (Fig. 21a). Length 3.03 mm. Color ferruginous. Body elongate-subovate, with distinct constriction between pronotum and elytron; in profile convex. Rostrum with median ridge and pair of submedian ridges ending in apical third; intervening furrows with rows of coarse punctures each containing one mesad directed narrow ochre scale; base dorsally protruding, projecting from profile subangularly; epistome posteriorly with indistinct irregular ridge. Forehead coarsely punctate-rugose. Pronotum with distinct subapical constriction; disk coarsely punctate-reticulate; with median costa; punctures each containing one narrow scale of ochre or white color. Elytra with striae deeply incised; intervals costate-carinate, with dense rows of punctures; punctures containing each one ochre or white narrow scale; base markedly bisinuate. Legs. Femora densely punctate, with sparse ochre scales, with transverse band of larger white scales. Profemur with subbasal callus anteriorly projecting. Tibiae subbasally with dorsal angulation; metatibia with suprauncal tooth. Abdominal ventrites 1-2 laterally swollen, medially forming common cavity, with coarse punctures; ventrite 5 medially concave, laterally swollen and with ochre elongate scales. Penis (Fig. 21b) with sides of body subparallel, subapically converging to subtruncate apex; endophallus with large X-shaped sclerite; complex transfer apparatus compact; ductus ejaculatorius without bulbus. **Intraspecific variation.** Length 2.58–3.19 mm. Body of females shorter. Female rostrum basally with median costa and pair of submedian costae; in apical half subglabrous, punctate; epistome simple. Female abdominal ventrites 1-2 medially flat; ventrite 5 flat.

##### Material examined.

Holotype (ANIC): ARC4045 (PCR failed), Queensland, Mt. Lewis Rd. via Julatten, 1000 m, rainforest, intercept trap, 11-XI-25-XII-1987. Paratypes (ANIC, SMNK): Queensland: 2 exx, same data as holotype; 1 ex, Mt. Lewis Rd. via Julatten, 1000 m, rainforest, intercept trap, 13-IX-10-X-1987; 1 ex, Mt. Lewis Rd. via Julatten, 1000 m, rainforest, intercept trap, 10-X-11-XI-1987; 1 ex, Mt. Lewis Rd. via Julatten, 01-XII-1975.

##### Distribution.

Queensland: Mt. Lewis Road.

##### Biology.

Sifted from leaf litter in primary forest.

##### Etymology.

This epithet is an adjective based on the name of the type locality, Mt. Lewis.

#### 22. Trigonopterus montanus

Taxon classificationAnimaliaColeopteraCurculionidae

Riedel sp. n.

http://zoobank.org/4652D7C5-8B0E-45F4-BFF6-4832E9A94CB8

##### Diagnostic description.

**Figure 22.**
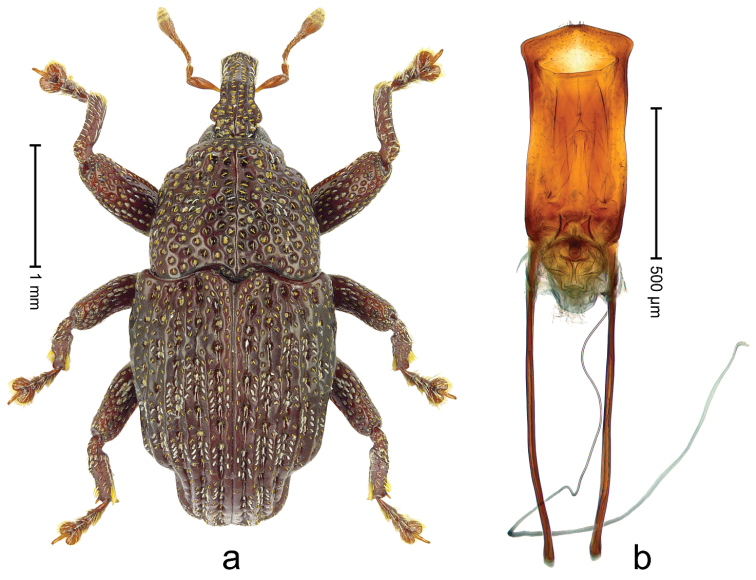
*Trigonopterus montanus* sp. n., holotype; **a** Habitus **b** Penis.

Holotype (Fig. 22a). Length 3.25 mm. Color ferruginous. Body elongate, with distinct constriction between pronotum and elytron; in profile convex. Rostrum with median ridge and pair of submedian ridges ending in apical third; intervening furrows with rows of coarse punctures each containing one mesad directed narrow scale; base dorsally protruding, gently projecting from profile; epistome posteriorly with 4 denticles. Forehead coarsely punctate-rugose. Pronotum with distinct subapical constriction; disk coarsely punctate-reticulate; with median costa; uneven, near middle with pair of weak swellings; punctures each containing one narrow scale of ochre or white color. Elytra with striae deeply incised, containing coarse punctures; intervals costate, punctate, in basal half partly transversely confluent; punctures containing small ochre scales or larger white scales; base markedly bisinuate. Legs. Femora densely punctate, with sparse scales. Profemur with subbasal callus anteriorly projecting. Tibiae subbasally denticulate; metatibia with suprauncal tooth. Abdominal ventrites 1-2 laterally swollen, medially forming common cavity, with coarse punctures; ventrite 5 in basal half concave, coarsely punctate. Penis (Fig. 22b) with sides of body subparallel, widened to subangulate apex; transfer apparatus compact, with pair of triangular sclerites; ductus ejaculatorius without bulbus. **Intraspecific variation.** Length 3.16–3.25 mm. Color ferruginous (ht, 1 pt), or black except tarsi and antenna ferruginous (1 pt). Female rostrum basally with median costa and pair of submedian costae; in apical half subglabrous, punctate; epistome simple. Female abdominal ventrites 1-2 medially flat; ventrite 5 flat.

##### Material examined.

Holotype (ANIC): ARC4040 (PCR failed), Queensland, Bellenden Ker Range, Summit TV Stn., S17°16', E145°51', 1560 m, rainforest, litter, Q.M. Berlesate No. 565, 29-IV-02-V-1983. Paratypes (ANIC, SMNK): Queensland: 2 exx, same data as holotype.

##### Distribution.

Queensland: Mt. Bellenden Ker.

##### Biology.

Sifted from leaf litter in primary forest.

##### Etymology.

This epithet is based on the adjective *montanus* (belonging to a mountain) and refers to the isolated occurrence of the species on the summit of Mt. Bellenden Ker.

#### 23. Trigonopterus monteithi

Taxon classificationAnimaliaColeopteraCurculionidae

Riedel sp. n.

http://zoobank.org/BF36E82F-CE3A-4310-934D-FCF8859275BA

##### Diagnostic description.

**Figure 23.**
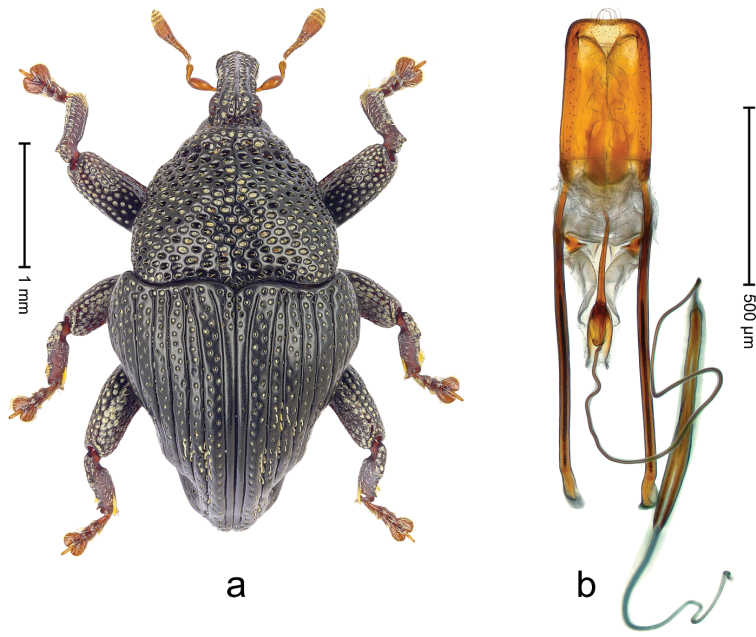
*Trigonopterus monteithi* sp. n., holotype; **a** Habitus **b** Penis.

Holotype (Fig. 23a). Length 3.22 mm. Color black; antenna and tarsi ferruginous. Body subrhomboid, with marked constriction between pronotum and elytron; in profile convex. Rostrum with median ridge and pair of submedian ridges; intervening furrows with rows of coarse punctures each containing one mesad directed seta; base dorsally protruding, markedly projecting from profile subangularly; epistome posteriorly with transverse ridge. Forehead coarsely punctate-rugose. Pronotum with sides converging to apex, almost without subapical constriction; foveate-reticulate; each fovea containing one inconspicuous seta; interspaces subglabrous, weakly microreticulate. Elytra cuneiform, from broad rounded humeri markedly converging to narrow apex; base bisinuate; surface microreticulate; striae deeply incised, narrow; intervals flat to weakly costate, with 1-2 rows of small punctures; sutural interval subglabrous except few punctures near base; intervals 2-5 behind middle with inconspicuous transverse patches of narrow recumbent scales. Legs. Femora densely punctate. Profemur with subbasal callus anteriorly projecting. Tibiae subbasally with dorsal angulation; pro- and metatibia subapically with suprauncal denticle. Abdominal ventrites 1-2 laterally swollen, medially forming common cavity; ventrite 5 coarsely punctate, weakly concave. Penis (Fig. 23b) with sides of body subparallel; apex subtruncate; endophallus with pair of subtriangular sclerites; transfer apparatus long spiniform; ductus ejaculatorius with distinct bulbus. **Intraspecific variation.** Length 2.04–3.53 mm. Female rostrum dorsally somewhat flattened; median ridge and pair of submedian costae subglabrous; epistome simple. Female abdominal ventrite 5 flat.

##### Material examined.

Holotype (QMBA): ARC3717 (EMBL # LN888199), Queensland, Kuranda, Saddle Mountain Road, S16°48.836', E145°39.580', sample 3, 586 m, 31-III-2014. Paratypes (QMBA, SMNK): Queensland: 1 ex, ARC3716 (EMBL # LN888198), same data as holotype; 2 exx, ARC3714 (EMBL # LN888196), ARC3715 (EMBL # LN888197), Kuranda, Saddle Mountain Road, S16°49.094', E145°39.712', sample 2, 637 m, 31-III-2014; 1 ex, 30 km N Kuranda, Black Mt. Rd., rainforest, leafmold, ANIC Berlesate 165, 04-XI-1969; 1 ex, Kuranda, Black Mt. Rd., S16°44', E145°34', 350 m, rainforest, ANIC Berlesate 339, 27-VI-1971; 1 ex, Davies Ck road, 20 km ESE Mareeba, 04-13-XII-88, 750 m, flight intercept trap; 1 ex, 9.6 km S Redlynch, Crystal Cascades, leaf litter, ANIC Berlesate 277, 30-IV-1970; 1 ex, Mt. Formartine South, 24-XI-1990, Berlesate 848, E145°37', S16°43', rainforest, 700 m, sieved litter; 2 exx, ARC3750 (EMBL # LN888230), ARC3751 (EMBL # LN888231), Daintree N.P., NW Mossman, Manjal Jimalji (Devils Thumb) trail, S16°23.571', E145°19.058', sample 2, 377 m, 20-IV-2014; 2 exx, ARC3746 (EMBL # LN888228), Cape Tribulation, Mt. Sorrow track, S16°04.789', E145°27.948', sample 1, 150 m, 09-IV-2014; 2 exx, ARC3745 (EMBL # LN888227), Cape Tribulation, Mt. Sorrow track, S16°04.579', E145°27.081', sample 6, 283 m, 10-IV-2014; 2 exx, ARC3747 (EMBL # LN888229), Cape Tribulation, Mt. Sorrow track, S16°04.695', E145°27.690', sample 7, 234 m, 10-IV-2014; 1 ex, 1,5 km NW of Cape Tribulation, site 1, S16°05', E145°28', Berlesate 480, 0 m, rainforest, sieved litter, 02-X-1982; 1 ex, 1,5 km NW of Cape Tribulation, site 1, Berlesate 445, S16°05', E145°28', 0 m, rainforest, sieved litter, 03-X-1982; 3 exx, 2.0 km W of Cape Tribulation, site 4, S16°05', E145°28', 200 m, Berlesate 429, sieved litter, rainforest, 25-IX-1982; 1 ex, 3.0 km W of Cape Tribulation, site 6, S16°05', E145°27', 500 m, Berlesate 422, rainforest, sieved litter, 19-IX-1982; 1 ex, 1.5 km W of Cape Tribulation, site 3, 150 m, baited flight trap, RF, 19-IX-1982; 2 exx, 2.5 km W of Cape Tribulation, site 5, S16°05', E145°27', 180 m, Berlesate 533, rainforest, sieved litter, 21-IV-1983; 1 ex, 2.5 km W of Cape Tribulation, site 5, S16°05', E145°27', 180 m, Berlesate 502, rainforest, sieved litter, 02-I-1983; 4 exx, Cape Tribulation, 49 km N of Daintree, 10 m, rainforest leaf and log litter, SBP 75, 12-VII-1982; 1 ex, Cape Tribulation, 49 km N of Daintree, 200 m, rainforest leaf litter, SBP 77, 14-VII-1982; 2 exx, Cape Tribulation, 10 m, rainforest streamside flood litter, SBP 79, 14-VII-1982; 1 ex, Table Mtn 10 km S of Cape Tribulation, S16°09', E145°26', 320 m, rainforest, sieved litter, 24-IV-1983; 1 ex, 2.0 km W of Cape Tribulation, site 4, S16°05', E145°28', 200 m, rainforest, sieved litter, 25-IX-1982; 1 ex, Cape Tribulation Area, S16°03', to S16°05', E145°28', 200 m, Winkler ANIC 1234 leaf and log litter, 01-11-V-1992; 3 exx, Thornton Range, S16°15', E145°26', 150 m, Berlesate ANIC 327, rainforest, 23-VI-1971; 2 exx, Thornton Range, S16°14', E145°26', 100 m, Berlesate ANIC 325, rainforest, 23-VI-1971; 1 ex, Thornton Range, S16°14', E145°26', 100 m, Berlesate ANIC 333, rainforest, 24-VI-1971; 4 exx, Thorn Rd., 11-XII-1969; 2 exx, Cooper Ck. near Daintree, S16°11', E145°26', 50 m, ANIC Berlesate 334, 22-VI-1971; 1 ex, Mt. Finnigan, 400 m, rainforest, litter and fungi, SPB56, 01-VII-1982; 1 ex, Mt. Finnigan, 400 m, rainforest, moist litter pockets, SPB61, 03-VII-1982; 1 ex, Moses Ck., 4 km NbyE of Mt. Finnigan, S15°47', E145°17', sieved rainforest litter, Berlesate ANIC 696, 14-16-X-1980; 3 exx, Julatten, edge of rainforest along creek, ex intercept trap, 21-30-XI-1987; 1 ex, Buchanan Ck., S 16°15', E145°26', 140 m, FIT B06F, 11-II-12-III-1998; 3 exx, Buchanan Ck., S 16°15', E145°26', 140 m, FIT B06F, 12-III-08-V-1998; 2 exx, Noah Beach, S 16°09', E145°26', 10 m, FIT N08F, 15-III-07-V-1998; 2 exx, Noah Beach, S 16°09', E145°26', 10 m, FIT N08F, 09-II-15-III-1998; 1 ex, Noah Beach, S 16°09', E145°26', 10 m, FIT N09F, 09-II-15-III-1998; 2 exx, Noah Beach, S 16°09', E145°26', 10 m, FIT N09F, 15-III-07-V-1998; 1 ex, Daintree, Buchanan Creek, S 16°14.39', E145°25.54', 140 m, FIT#6, 15-I-11-II-1998; 2 exx, Daintree, Cooper Creek, S 16°09.10', E145°24.19', 140 m, FIT#9, 11-I-10-II-1998; 2 exx, Daintree, Cooper Creek, S 16°09', E145°24', 140 m, FIT C05F, 16-III-07-V-1998; 2 exx, Daintree, Thompson Creek, S 16°07', E145°25', 80 m, FIT T08F, 10-I-12-II-1998; 1 ex, Daintree, Thompson Creek, S 16°07', E145°25', 80 m, FIT T09F, 12-II-15-III-1998; 2 exx, Daintree, Thompson Creek, S 16°07', E145°25', 80 m, FIT T09F, 15-III-07-V-1998; 1 ex, Daintree, Thompson Creek, S 16°07', E145°25', 80 m, FIT T09F, 10-I-12-II-1998; 10 exx, Daintree, Pimm´s Block, S 16°11', E145°24', 100 m, FIT T07F, 13-III-08-V-1998; 1 ex, Daintree, Pimm´s Block, S 16°11.33', E145°24.30', 100 m, FIT =6, 08-I-09-II-1998; 1 ex, Hutchinson Ck., S 16°13', E145°24', 30 m, FIT H09F, 11-II-14-III-1998; 4 exx, Hutchinson Ck., S 16°13', E145°24', 30 m, FIT H09F, 14-III-08-V-1998; 2 exx, Donovan Ck., S 16°01', E145°27', 20 m, FIT D09F, 10-II-14-III-1998; 3 exx, Donovan Ck., S 16°01', E145°27', 20 m, FIT D07F, 14-III-06-V-1998; 1 ex, Fairy Ck., S 16°14', E145°25', 80 m, FIT F07F, 11-II-13-III-1998; 1 ex, Fairy Ck., S 16°14', E145°25', 80 m, FIT F07F, 12-I-11-II-1998; 1 ex, Fairy Ck., S 16°14', E145°25', 80 m, FIT F01F, 13-III-08-V-1998; 4 exx, ARC3759 (EMBL # LN888236), ARC3760 (EMBL # LN888237), ARC3761 (EMBL # LN888238), Cedar Bay N.P., road between Rossville and Bloomfield, S15°47.510', E145°18.141', sample 2, 322 m, 29-IV-2014; 4 exx, Cedar Bay N.P., road between Rossville and Bloomfield, S15°47.510', E145°18.141', sample 2-B, 322 m, 01-V-2014; 3 exx, Cedar Bay N.P., road between Rossville and Bloomfield, S15°48.274', E145°18.901', sample 4, 214 m, 29-IV-2014; 1 ex, 3 km NE Mt. Webb, S15°03', E145°09', 01-30-X-1980.

##### Distribution.

Queensland: Cairns, Kuranda, Lamb Range, Mt. Formartine South, Cape Tribulation, Daintree N.P., Cedar Bay N.P., Mt. Webb.

##### Biology.

Sifted from leaf litter in primary forest.

##### Etymology.

This species is named in honor of Geoff Monteith (Brisbane), who collected the majority of the new Australian *Trigonopterus* species for the first time and whose help was essential for the success of this study.

##### Notes.

*Trigonopterus monteithi* Riedel, sp. n. was coded as “*Trigonopterus* sp. 553”.

#### 24. Trigonopterus mossmanensis

Taxon classificationAnimaliaColeopteraCurculionidae

Riedel sp. n.

http://zoobank.org/6B1EA8BB-66D3-437D-9F5B-F27F75DDD450

##### Diagnostic description.

**Figure 24.**
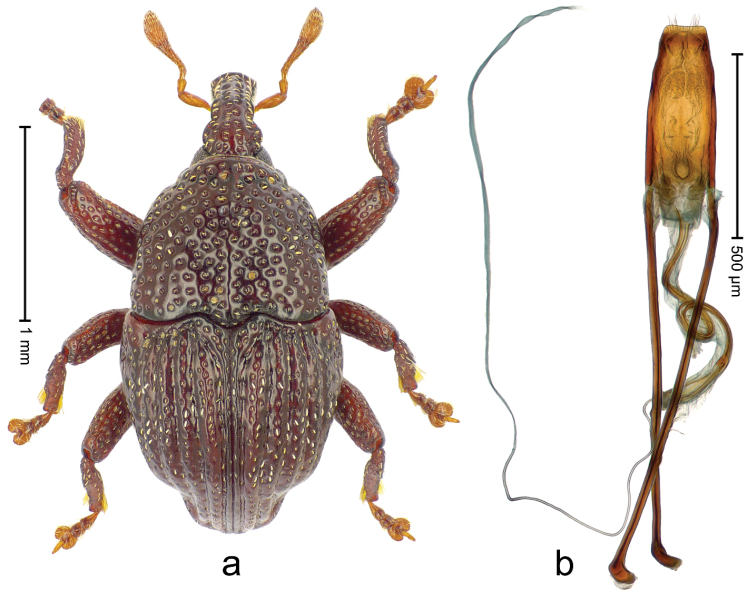
*Trigonopterus mossmanensis* sp. n., holotype; **a** Habitus **b** Penis.

Holotype (Fig. 24a). Length 1.92 mm. Color ferruginous. Body subovate, with weak constriction between pronotum and elytron; in profile convex. Rostrum with 4 rows of coarse punctures each containing one mesad directed seta; without distinct ridges; base gently projecting from profile; epistome indistinct. Forehead coarsely punctate. Pronotum with indistinct subapical constriction; disk coarsely punctate; punctures each containing one inconspicuous seta, very few with yellow recumbent scales instead. Elytra with striae deeply incised; intervals costate, punctate, with scattered recumbent scales; sutural interval narrow and weakly convex, below level of interval 2; base markedly bisinuate. Legs. Femora densely punctate. Profemur with subbasal callus anteriorly projecting. Tibiae subbasally with dorsal angulation; uncus of protibia slender, hook-shaped, basally continued with ventral tibial outline, markedly curved ventrad towards apex. Abdominal ventrites 1 laterally swollen, medially concave; abdominal ventrite 2 swollen; ventrite 5 microgranulate, weakly concave. Penis (Fig. 24b) with sides of body converging, apex subtruncate; transfer apparatus flagelliform, ca. 1.8× longer than body of penis; ductus ejaculatorius without bulbus. **Intraspecific variation.** Length 1.92–2.11 mm. Color ferruginous or black with only legs and head ferruginous. Female rostrum dorsally somewhat flattened; with median costa and pair of submedian costae; epistome simple. Female abdominal ventrites 1-2 medially flat; ventrite 5 coarsely punctate.

##### Material examined.

Holotype (QMBA): ARC3896 (PCR failed), Queensland, Mossman Bluff Track, 5-10 km W Mossman, site 6, 860m, flt. Intercept, 16-30-XII-1988. Paratype (QMBA) 1 ex, Mossman Bluff Track, 5-10 km W Mossman, site 7, 1000 m, pitfall, 16-30-XII-1988.

##### Distribution.

Queensland: Mossman Bluff.

##### Biology.

Sifted from leaf litter in primary forest.

##### Etymology.

This epithet is an adjective based on the name of the type locality, Mossman.

#### 25. Trigonopterus oberprieleri

Taxon classificationAnimaliaColeopteraCurculionidae

Riedel sp. n.

http://zoobank.org/9E38DA44-1AD6-45FE-ACE9-FC4679E643D3

##### Diagnostic description.

**Figure 25.**
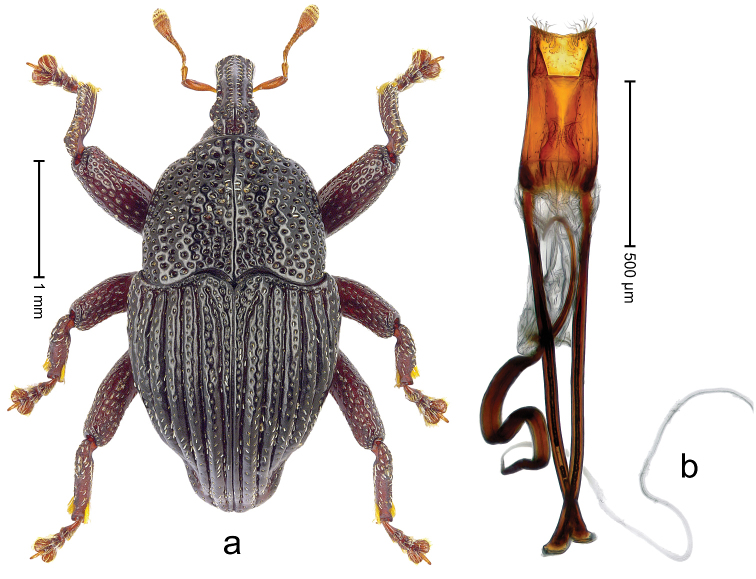
*Trigonopterus oberprieleri* sp. n., holotype; **a** Habitus **b** Penis.

Holotype (Fig. 25a). Length 3.11 mm. Color black; antenna and legs ferruginous. Body subovate, with marked constriction between pronotum and elytron; in profile convex. Rostrum with median costa and pair of submedian costae; intervening furrows with rows of coarse punctures each containing one mesad directed seta; base dorsally protruding, markedly projecting from profile subangularly; epistome posteriorly with irregular ridge. Forehead coarsely punctate-rugose. Pronotum with subapical constriction; disk coarsely punctate; with median costa, bordered by pair of submedian longitudinal impressions. Elytra with striae deeply incised; intervals costate, microreticulate, punctate, with scattered recumbent white scales; base markedly bisinuate. Legs. Femora densely punctate. Profemur with subbasal callus anteriorly projecting. Tibiae subbasally with dorsal angulation; metatibia in apical third with blunt suprauncal projection. Metaventrite and abdominal ventrites 1-2 laterally swollen, medially forming common cavity; metaventrite and abdominal ventrite 1 with dense erect setae, abdominal ventrite 2 with erect scales; ventrite 5 punctate, weakly concave. Penis (Fig. 25b) with sides of body subparallel, apex subtruncate, with weak median incision; transfer apparatus flagelliform, curved ventrad, subequal to body of penis; ductus ejaculatorius near insertion to transfer apparatus sclerotized, forming S-shaped ribbon longer than flagellum; without bulbus. **Intraspecific variation.** Length 2.55–3.14 mm. Female body more slender. Female rostrum dorsally somewhat flattened; median costa and pair of submedian costae subglabrous; epistome simple. Female abdominal ventrites 1-2 medially flat; ventrite 5 punctate, concave.

##### Material examined.

Holotype (QMBA): ARC3742 (EMBL # LN888224), Queensland, Cape Tribulation, Mt. Sorrow track, S16°04.579', E145°27.081', sample 6, 283 m, 10-IV-2014. Paratypes (QMBA, SMNK): Queensland: 2 exx, ARC3743 (EMBL # LN888225), ARC3744 (EMBL # LN888226), same data as holotype; 3 exx, Cape Tribulation, Mt. Sorrow track, S16°04.491', E145°26.873', sample 3, 343 m, 10-IV-2014; 1 ex, ARC3767 (EMBL # LN888244), 2,5 km W Mt. Hartley, near Rossville – Bloomfield-road, S15°47.393', E145°18.348',sample 3, 419 m, 01-V-2014; 1 ex, Daintree N.P.,Thompson Ck., S16°07', E145°25', FIT T01f, 10-I-12-II-1998.

##### Distribution.

Queensland: Daintree N.P., Surroundings of Mt. Hartley.

##### Biology.

Sifted from leaf litter in primary forest.

##### Etymology.

This species is named in honor of Rolf Oberprieler, who made available for study the many *Trigonopterus* specimens in the ANIC.

##### Notes.

*Trigonopterus oberprieleri* Riedel, sp. n. was coded as “*Trigonopterus* sp. 557”.

#### 26. Trigonopterus robertsi

Taxon classificationAnimaliaColeopteraCurculionidae

Riedel sp. n.

http://zoobank.org/AB656BB2-7AB2-493B-B9E9-B39442D750DB

##### Diagnostic description.

**Figure 26.**
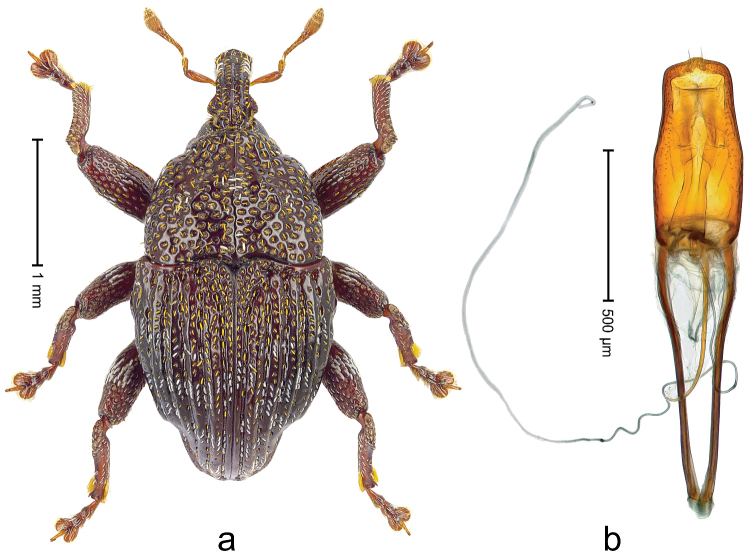
*Trigonopterus robertsi* sp. n., holotype; **a** Habitus **b** Penis.

Holotype (Fig. 26a). Length 2.88 mm. Color ferruginous. Body subovate, with distinct constriction between pronotum and elytron; in profile convex. Rostrum with median ridge and pair of submedian ridges ending in apical third; intervening furrows with rows of coarse punctures each containing one mesad directed narrow scale; base dorsally protruding, markedly projecting from profile subangularly; epistome posteriorly with subangulate ridge. Forehead coarsely punctate-rugose. Pronotum with distinct subapical constriction; disk coarsely punctate-reticulate; with median costa; uneven, with weak swelling at center of disk; punctures each containing one narrow ochre scale. Elytra with striae deeply incised; intervals costate-carinate; with rows of narrow ochre scales and larger white scales; base markedly bisinuate. Legs. Femora densely punctate, with sparse ochre scales, with transverse band of larger white scales. Profemur with subbasal callus anteriorly projecting. Tibiae subbasally with dorsal angulation; metatibia with suprauncal tooth. Abdominal ventrites 1-2 laterally swollen, medially forming common cavity, with coarse punctures; ventrite 5 weakly concave, in basal half with ochre scales. Penis (Fig. 26b) with sides of body subparallel, with constriction in front of middle, continued subparallel to subangulate apex; transfer apparatus flagelliform, directed basad, its length subequal to body of penis; ductus ejaculatorius without bulbus. **Intraspecific variation.** Length 2.58–3.08 mm. Color ferruginous or almost black with only tarsi and antenna ferruginous. Female rostrum with median costa and pair of submedian costae; epistome simple. Female abdominal ventrites 1-2 medially flat; ventrite 5 flat.

##### Material examined.

Holotype (QMBA): ARC3727 (EMBL # LN888209), Queensland, Mt. Finnigan, ascent from Shiptons Flat, S15°49.001', E145°16.853', 1075 m, sample 1, 28-IV-2014. Paratypes (QMBA, SMNK): Queensland: 5 exx, ARC3728 (EMBL # LN888210), ARC3729 (EMBL # LN888211), ARC3730 (EMBL # LN888212), same data as holotype; 1 ex, Mt. Finnigan, ascent from Shiptons Flat, S15°48.935', E145°16.669', 1048 m, sample 2, 28-IV-2014; 1 ex, Mt. Finnigan, site 4, S15°48', E145°17',1060 m, pitfalls, 04-XII-1990-17-I-1991; 1 ex, Finnigan summit, S15°49', E145°17', 1100 m, rainforest, stick brushing, Berlesate 979, 21-XI-1998; 1 ex, 4,0 km W of Cape Tribulation, site 8, 720 m, rainforest pitfall traps, sieved litter, 23-IX-7-X-1982; 1 ex, Mt. Hartley summit, S15°46', E145°19', 790 m, intercept trap, 08-XI-1995-16-I-1996; 2 exx, Mt. Hartley summit, S15°46', E145°19', 750 m, pitfall traps, 08-XI-1995-16-I-1996.

##### Distribution.

Queensland: W Cape Tribulation, Mt. Finnigan, Mt. Hartley.

##### Biology.

Sifted from leaf litter in primary forest.

##### Etymology.

This species is named in honor of the naturalist Lewis Roberts (Shiptons Flat), whose guiding help was essential for the discovery of this species.

##### Notes.

*Trigonopterus robertsi* Riedel, sp. n. was coded as “*Trigonopterus* sp. 556”.

#### 27. Trigonopterus rostralis

Taxon classificationAnimaliaColeopteraCurculionidae

(Lea)

Idotasia rostralis Lea, 1928: 155–156.
Trigonopterus rostralis (Lea): Pullen et al. 2014: 271.

##### Diagnostic description.

**Figure 27.**
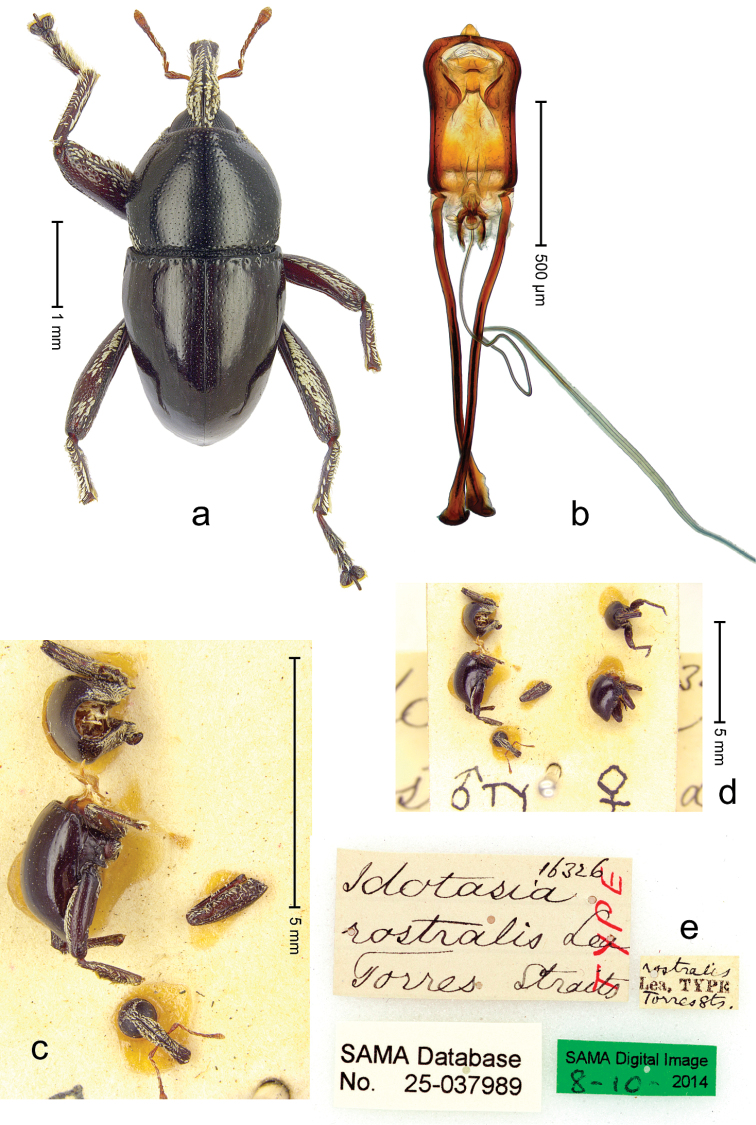
*Trigonopterus rostralis* (Lea), male lectotype; **a** Habitus **b** Penis **c** as mounted originally **d** original labels.

Lectotype (Fig. 27a). Length 3.81 mm. Color black; legs and antenna ferruginous. Body subovate; in dorsal aspect and in profile with constriction between pronotum and elytron. Rostrum slender, dorsally with glabrous median carina; in basal half clothed with mesad directed, white, spatulate scales; subapically subglabrous, punctate, sparsely setose. Eyes large, in dorsal position. Pronotum large; disk separated from sides by distinct edge; disk subglabrous, densely punctate with minute punctures, posterolateral and anterolateral corner clothed with white scales; sides in anterior 1/3 clothed with white scales. Elytra subglabrous, punctate with minute punctures; basal margin bordered by row of large punctures continued behind humeri. Legs. Profemur large; anterior face with white scales. Mesofemur and metafemur dorsally densely squamose with white scales, with distinct dorsoposterior edge. Abdominal ventrite 5 flat. Aedeagus (Fig. 27b) with body in basal half subparallel, widened in apical half; extended into acute median process; ductus ejaculatorius subapically with weak bulbus. **Intraspecific variation.** Length 3.22–3.81 mm. Female rostrum dorsally largely subglabrous, with sublateral rows of minute punctures; in basal 1/5 punctate-rugose, sparsely clothed with white scales.

##### Material examined.

Type specimens. Male, lectotype by present designation (SAMA): Queensland, Torres Straits (labels Fig. 27e), ARC4037 (PCR failed). Female, paralectotype (SAMA), same data as lectotype. Other specimens (QMBA, SAMA, SMNK): Queensland: 1 ex, 3 km ENE of Mt.Tozer, S12°44', E143°14', Malaise trap; 28-VI-04-VII-1986; 4 exx, 11 km ENE of Mt.Tozer, S12°43', E143°18', 5-10-VII-1986; 1 ex, 9 km ENE of Mt.Tozer, S12°43', E143°17', swept from undergrowth, 5-10-VII-1986; 4 exx, 9 km ENE of Mt.Tozer, S12°43', E143°17', 11-16-VII-1986; 2 exx, 9 km ENE of Mt.Tozer, S12°43', E143°17', Malaise trap, 11-16-VII-1986; 1 ex, 11 km ENE of Mt.Tozer, S12°43', E143°18', swept from undergrowth, 11-16-VII-1986; 1 ex, 11 km ENE of Mt.Tozer, S12°43', E143°18', ex yellow trays, 11-16-VII-1986; 2 exx, Claudie River, 4 km SW road junction, S12°44', E143°15', 04-XII-1986.

##### Distribution.

Queensland: Torres Strait, Iron Range N.P.

##### Biology.

Swept and beaten from forest undergrowth.

##### Notes.

Lea’s (1928) description is based on a male and a female specimen, and although Lea marked the male with a handwritten “TY” on its card, as he usually did to indicate the specimen he regarded to be the type, he did not designate it as the holotype in his description. This male is here designated as lectotype to ensure stability of nomenclature in case additional syntypes are discovered that belong to different species.

#### 28. Trigonopterus sculptirostris

Taxon classificationAnimaliaColeopteraCurculionidae

(Lea)

Idotasia sculptirostris Lea, 1928: 154–155.
Trigonopterus sculptirostris (Lea): Pullen et al. 2014: 271.

##### Diagnostic description.

**Figure 28.**
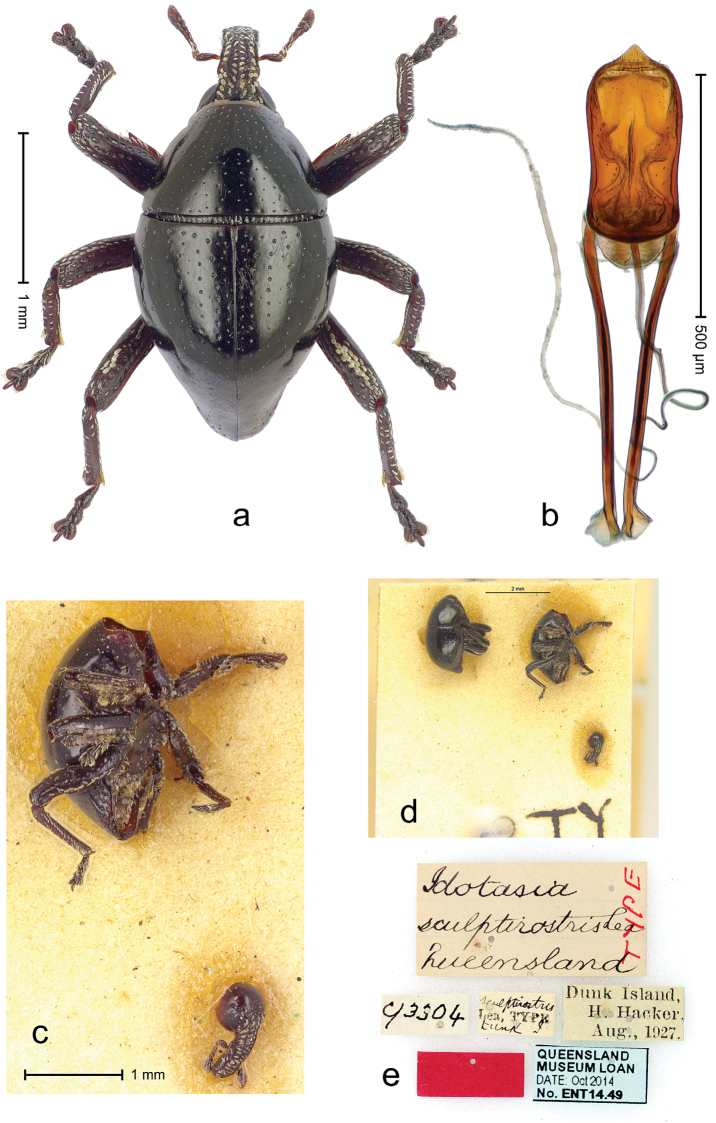
*Trigonopterus sculptirostris* (Lea), male lectotype; **a** Habitus **b** Penis **c** as mounted originally **d** original labels.

Lectotype (Fig. 28a). Length 2.24 mm. Color black. Body subovate, almost without constriction between pronotum and elytron; in profile evenly convex. Rostrum with sharp median ridge and pair of sharp submedian ridges; intervening furrows with rows of white scales; apical 1/3 rugose-punctate. Eyes with dorsal margin bordered by furrow. Forehead punctate. Pronotum with disk densely punctate with small punctures; sides sparsely shallowly foveate. Elytra subglabrous, striae marked rows of small punctures; humeri laterally with row of large punctures. Legs with sparse white scales; anteroventral ridge of pro- and mesofemur with acute tooth, metafemur with blunt tooth. Metafemur dorsally with sparse white scales; posterior surface with furrow containing row of scales parallel to ventral edge, subdorsally with row of coarse punctures. Tibial apex with uncus, without premucro. Abdominal ventrite 2 posteriorly costate resembling ventrite 3, anteriorly declivous to concave ventrite 1; ventrite 5 weakly concave, punctate, sparsely clothed with erect scales and setae. Penis (Fig. 28b) with sides of body subparallel, weakly concave; apex with median triangular extension confluent with outline of apex; transfer apparatus short, dentiform, supported by lyriform sclerite; ductus ejaculatorius without bulbus. **Intraspecific variation.** Length 2.14–2.80 mm. Female rostrum with dorsal ridges less distinct, only in basal half; apical 1/2 rugose-punctate. Female abdominal ventrites 1 and 2 medially flat; female abdominal ventrite 5 flat.

##### Material examined.

Type specimens. Male, lectotype by present designation (Fig. 28) (QMBA): Queensland, Dunk Island, coll. H. Hacker, VIII-1927 (labels Fig. 28e), ARC4039 (PCR failed). Female, paralectotype (SAMA), same data as lectotype. Other specimens (ANIC, SMNK): Queensland: 10 exx, ARC3669 (EMBL # LN888170), ARC3670 (EMBL # LN888171), ARC3671 (EMBL # LN888172), Kuranda N.P., Saddle Mountain Road, S16°48.882', E145°38.870', to S16°48.559', E145°39.458', 380–475 m, 31-III-2014; 40 exx, ARC3681 (EMBL # LN888173), ARC3682 (EMBL # LN888174), ARC3683 (EMBL # LN888175), ARC3684 (EMBL # LN888176), Mission Beach, Clump Mt. N.P., Bicton Hill, S17°50.146', E146°06.023', to S17°50.499', E146°05.905', 36-240 m, 14-IV-2014; 5 exx, ARC3687 (EMBL # LN888177), ARC3688 (EMBL # LN888178), Djiru N.P., road between Mission Beach and El Arish, S17°52.053', E146°04.093', 75 m, 15-IV-2014; 2 exx, Julatten, edge of rainforest along creek, ex intercept trap, 20-X-21-XI-1987; 20 exx, ARC3756 (EMBL # LN888233), ARC3757 (EMBL # LN888234), ARC3758 (EMBL # LN888235), Daintree N.P., NW Mossman, Manjal Jimalji (Devils Thumb) trail, S16°23.653', E145°19.724', to S16°23.664', E145°18.531', 100-700 m, 20-IV-2014; 1 ex, Donovan Ck., S16°01', E145°27', 20 m, FIT D03F, 10-II-14-III-1998; 1 ex, ARC3861 (EMBL # LN888245), Mt. Finnigan, ascent from Shiptons Flat, S15°48.620', E145°16.329', to S15°49.043', E145°16.780', 700-1000 m, 28-IV-2014.

##### Distribution.

Queensland: Mission Beach, Dunk Island; Kuranda, Julatten, Daintree N.P., Mt. Finnigan.

##### Biology.

Beaten from foliage in rainforest.

##### Notes.

Lea (1928) did not designate a holotype in the original description nor specify the number of specimens examined. The original description is based on more than one specimen. One pair with the male marked “TY” could be examined but other specimens may exist. The male is here designated lectotype to ensure stability of nomenclature in case additional syntypes are discovered that belong to different species.

#### 29. Trigonopterus squamosus

Taxon classificationAnimaliaColeopteraCurculionidae

(Lea)

Idotasia squamosa Lea, 1928: 155.
Trigonopterus squamosus (Lea): Zimmerman 1992: 376.

##### Diagnostic description.

**Figure 29.**
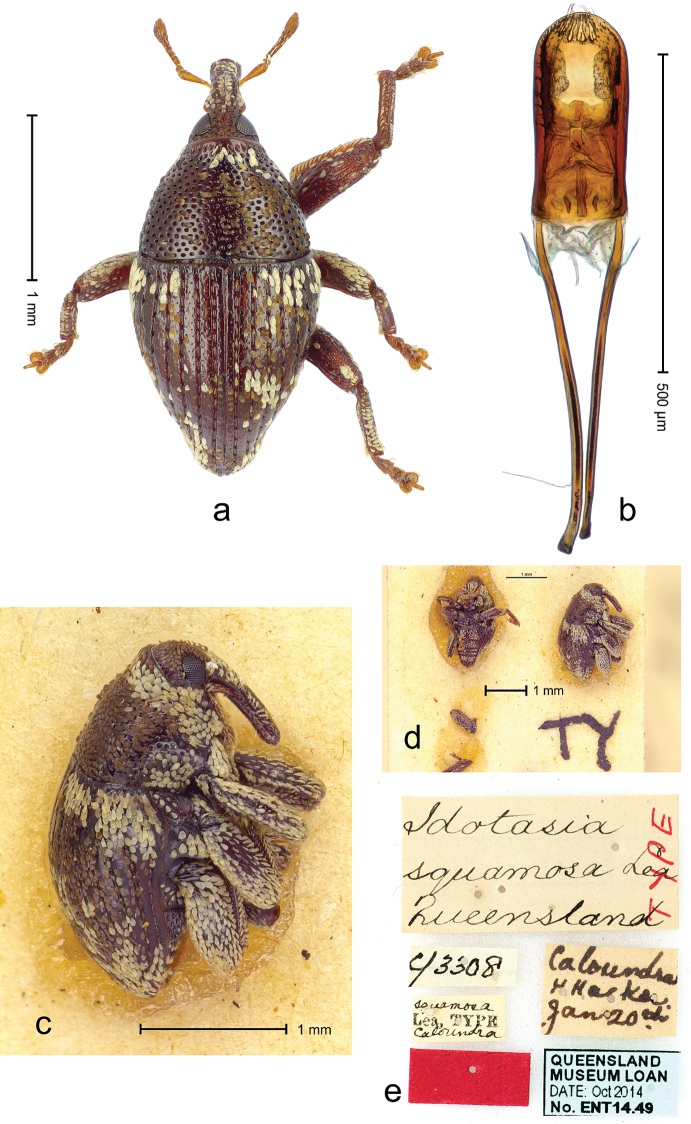
*Trigonopterus squamosus* (Lea), male lectotype; **a** Habitus **b** Penis **c** paralectotype **d** as mounted originally **e** original labels.

Lectotype (Fig. 29a). Length 2.10 mm. Color ferruginous; integument partly covered with brown or white scales, partly abraded. Body subrhomboid, with weak constriction between pronotum and elytron; in profile evenly convex. Rostrum with median ridge and pair of less distinct submedian ridges; covered with white scales. Eyes large, in subdorsal position. Forehead punctate, covered with brown scales. Pronotum coarsely punctate, covered with scales inserting at punctures, interspaces polished; disk clothed with brown scales, laterally and subapically with white scales. Elytra with striae deeply incised, narrow; intervals flat, each with two rows of scales largely covering surface, sutural interval with only one row; abraded scales leaving small punctures at point of insertion; subbasally and subapically clothed with white scales, remainder with brown scales and sparse white scales. Legs. Fore- and hind leg broken off and glued separately to card; largely covered with white scales except subglabrous posterior face of meso- and metafemur. Profemur with anteroventral ridge basally abruptly ending forming blunt angle; with subovate, slightly concave subbasal callus. Tibial apex with uncus and minute premucro. Abdominal ventrite 1-2 laterally swollen, medially concave. Penis (Fig. 29b) with sides of body subparallel to rounded apex; transfer apparatus simple, spiniform, supported by pair of small elongate sclerites; ductus ejaculatorius without bulbus. **Female paralectotype**: Length 2.40 mm. Body wider, rather subovate. Rostrum in apical half subglabrous, with sublateral sparse rows of scales. Abdominal ventrites 1 and 2 medially flat. **Intraspecific variation.** Length 2.00–2.40 mm.

##### Material examined.

Type specimens. Male, lectotype by present designation (QMBA): Queensland, Caloundra, coll. H. Hacker, 20-I (labels Fig. 29e), ARC4036 (PCR failed). Female, paralectotype (SAMA), ARC4035 (PCR failed), same data as lectotype. Other specimens (QMBA, SMNK): Queensland: 7 exx, Fraser Isl., Lake Allom, S25°11', E153°13', ANZES Exped., XI-1992.

##### Distribution.

Queensland: Caloundra, Fraser Isl..

##### Biology.

Beaten from foliage of undergrowth in relatively dry forest.

##### Notes.

Lea (1928) did not designate a holotype in the original description nor specify the exact number of specimens examined. One pair with the female marked “TY” could be examined but other specimens may exist. The male syntype is here designated lectotype. The diagnosis of this species is difficult, and E. C. Zimmerman (unpublished note in QMBS) and Pullen et al. (2014) considered its name to be synonymous with that of *Trigonopterus striatipennis* (Lea). However, specimens collected at one locality of North Stradbroke Island fall into two highly divergent clusters based on CO1 sequences. These sequence clusters are correlated with relatively subtle differences in the male genitalia. One is identical to the species described from North Stradbroke Island by Lea (1928), i.e. *Trigonopterus striatipennis*; the other is close to *Trigonopterus squamosus*. There remains some uncertainty whether all populations of this complex belong to the same two sibling species or if additional cryptic species exist. Sequence data from specimens of additional localities need to be analyzed for a final clarification. The specimen illustrated by Zimmerman (1992, p. 377, plate 492) shows a specimen of *Trigonopterus striatipennis* Lea.

#### 30. Trigonopterus striatipennis

Taxon classificationAnimaliaColeopteraCurculionidae

(Lea) comb. n.

Idotasia striatipennis Lea, 1928: 155.

##### Diagnostic description.

**Figure 30:**
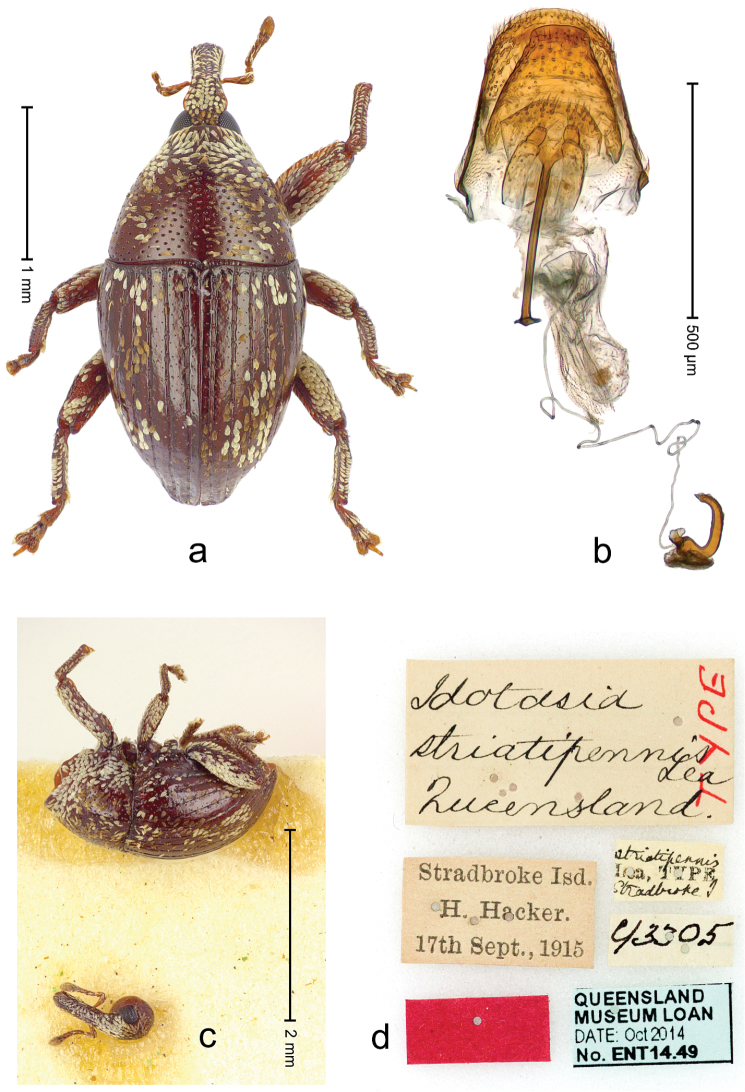

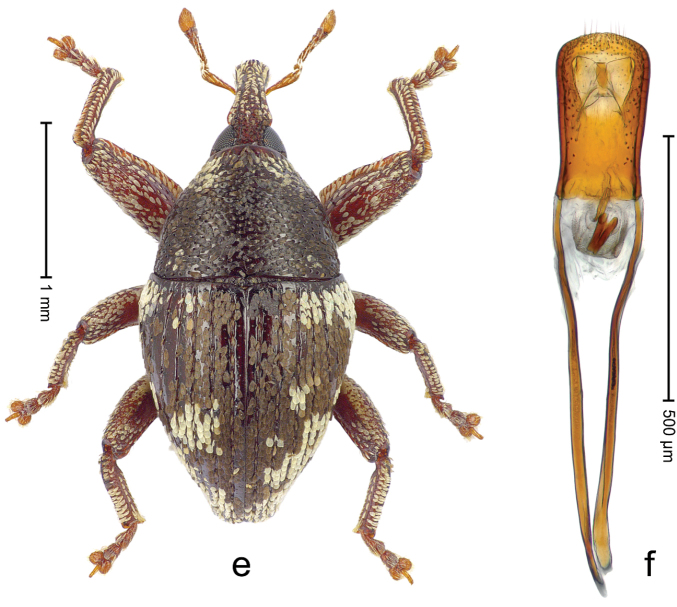
*Trigonopterus striatipennis* (Lea), female holotype; **a** Habitus **b** terminalia **c** as mounted originally **d** original labels. *Trigonopterus striatipennis* (Lea), male; **e** Habitus **f** Penis.

Holotype (Fig. 30a). Length 2.43 mm. Color ferruginous; integument partly covered with brown or white scales, largely abraded. Body subovate; with weak constriction between pronotum and elytron; in profile evenly convex. Rostrum in apical half with submedian rows of punctures, sparsely covered with white scales. Eyes large, in subdorsal position. Forehead punctate, covered with brown scales. Pronotum coarsely punctate, covered with scales inserting at punctures, interspaces polished; disk clothed with brown scales, laterally and subapically with white scales. Elytra with striae deeply incised, narrow; intervals flat, each with two rows of scales largely covering surface, sutural interval with only one row; abraded scales leaving small punctures at point of insertion; subbasally and subapically clothed with white scales, remainder with brown scales and sparse white scales. Legs. Left foreleg broken off and missing; largely covered with white scales except subglabrous posterior face of meso- and metafemur and where abraded. Profemur with anteroventral ridge basally abruptly ending forming blunt angle; with subovate, slightly concave subbasal callus. Tibial apex with uncus, without premucro. Abdominal ventrites 1-2 medially flat. Terminalia (Fig. 30b). **Male** (ARC3663, Fig. 30e). Male rostrum with median ridge and pair of submedian ridges; covered with white scales. Abdominal ventrites 1-2 laterally swollen, medially concave. Penis (Fig. 30f) with sides of body slightly diverging to widened, rounded apex; transfer apparatus simple, spiniform, supported by single Y-shaped sclerite; ductus ejaculatorius without bulbus. **Intraspecific variation.** Length 2.32–2.53 mm. One specimen (ARC3666) with conspicuous pair of protrusions behind eyes, apparently a rare aberration.

##### Material examined.

Type specimens. Female, holotype by monotypy (QMBA): Queensland, Stradbroke Island, coll. H. Hacker, 17-IX-1915 (labels Fig. 30d), ARC4034 (PCR failed). Other specimens (ANIC, QMBA, SMNK): Queensland: 5 exx, ARC3663 (EMBL # LN888167), ARC3665 (EMBL # LN888168), ARC3666 (EMBL # LN888169), North Stradbroke Isl., 3,5 km SW Point Lookout, Fishermans Road, 105 m, S27°26.507', E153°30.353', 24-III-2014, beaten from forest understorey; 8 exx, North Stradbroke Isl., track to Blue Lake, on *Pteridium*, 11-IX-1984; 7 exx, North Stradbroke Isl., track to Blue Lake, on *Pteridium*, 02-IX-1983; 3 exx, N Stradbroke Isl. Enterprise, S27°33', E153°28', Blackbutt #1, 90 m, 09-I-2002, sweeping 50934.

##### Distribution.

Queensland: North Stradbroke Island.

##### Biology.

Beaten from foliage of undergrowth in relatively dry forest.

##### Notes.

Lea (1928) stated in his description that the “type” was a “unique” specimen, and it therefore has to be regarded as the holotype. Regarding the distinction of this species from *Trigonopterus squamosus* (Lea), see the remarks above.

#### 31. Trigonopterus terraereginae

Taxon classificationAnimaliaColeopteraCurculionidae

Riedel sp. n.

http://zoobank.org/C801E410-1A73-4BBA-BDB1-60706499D224

##### Diagnostic description.

**Figure 31.**
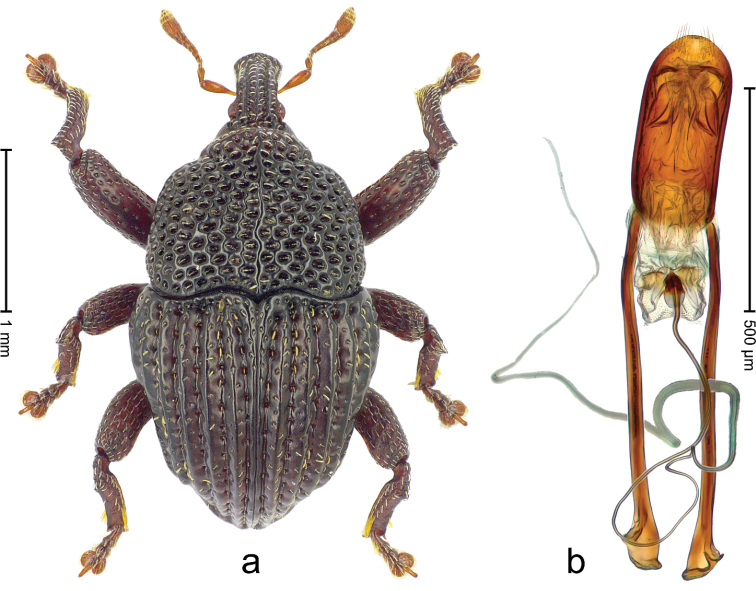
*Trigonopterus terraereginae* sp. n., holotype; **a** Habitus **b** Penis.

Holotype (Fig. 31a). Length 2.50 mm. Color black; antenna and legs ferruginous. Body subovate, in dorsal aspect with marked constriction between pronotum and elytron; in profile convex. Rostrum with median ridge and pair of submedian ridges; intervening furrows with rows of coarse punctures each containing one mesad directed scale; epistome posteriorly with curved ridge. Forehead coarsely punctate-rugose. Pronotum with sides subparallel, anteriorly abruptly rounded to distinct subapical constriction; irregularly foveate-reticulate; each fovea containing one inconspicuous seta. Elytra with striae deeply incised, with coarse punctures; intervals costate-carinate; subglabrous, sparsely punctate, with sparse scales; base bisinuate. Legs. Femora densely punctate. Profemur with subbasal callus anteriorly projecting. Tibiae subbasally with acute tooth; metatibia with suprauncal tooth. Abdominal ventrite 1 concave; abdominal ventrite 2 posteriorly transversely costate. Penis (Fig. 31b) with sides of body subparallel, apex subangulate, medially rounded; orifice with pair of curved sclerites; transfer apparatus short, dentiform; ductus ejaculatorius subapically with weak bulbus.

##### Material examined.

Holotype (ANIC): ARC4242 (PCR failed), Queensland, Mt. Cook N.P., S15°29', E145°16', rainforest litter, ANIC Berlesate No. 732, 10-12-V-1981.

##### Distribution.

Queensland: Mt. Cook.

##### Biology.

Sifted from leaf litter in primary forest.

##### Etymology.

This epithet refers to Queensland (*Terra Reginae*).

#### 32. Trigonopterus yorkensis

Taxon classificationAnimaliaColeopteraCurculionidae

Riedel sp. n.

http://zoobank.org/2D448C5C-AA72-486A-BF59-2AE27383D75E

##### Diagnostic description.

**Figure 32.**
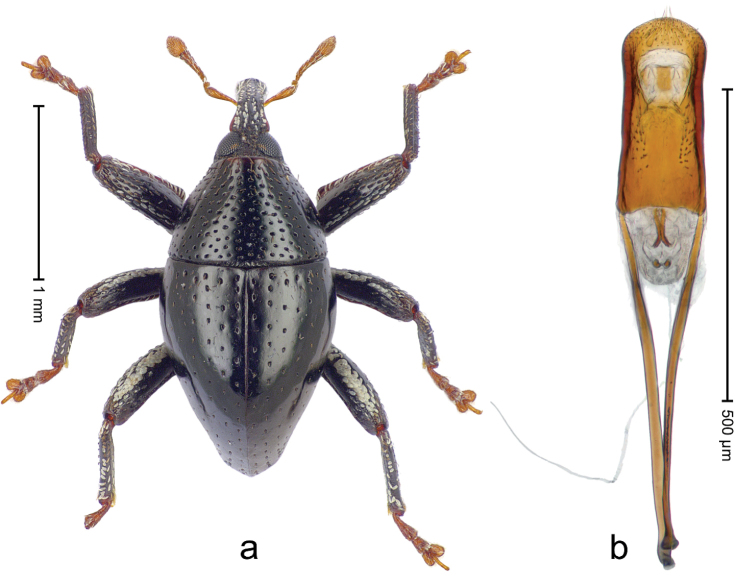
*Trigonopterus yorkensis* sp. n., holotype; **a** Habitus **b** Penis.

Holotype (Fig. 32a). Length 1.84 mm. Color black, antenna and tarsi ferruginous. Body subrhomboid, without constriction between pronotum and elytron; in profile evenly convex. Rostrum punctate-rugose, with sparse white scales, median ridge indistinct. Eyes large, in subdorsal position. Forehead punctate, with scattered brown scales. Pronotum coarsely punctate, interspaces between punctures polished; with sparse narrow brown scales inserting in punctures. Elytra subglabrous; striae marked by indistinct rows of minute punctures each containing minute narrow brown scale; at apical margin with few white almond-shaped scales. Legs. Femora dorsally clothed with white scales; anteroventral furrow with sparse row of white scales. Profemur with anteroventral ridge basally abruptly ending forming blunt angle; with somewhat indistinct subbasal callus. Tibial apex with uncus, without premucro. Abdominal ventrites 1-2 laterally swollen, medially concave; ventrite 5 coarsely punctate, at middle concave. Penis (Fig. 32b) with sides of body subparallel to subangulate apex; transfer apparatus simple, elongate, with pair of small basal sclerites; ductus ejaculatorius without bulbus. **Intraspecific variation.** Length 1.70–1.96 mm. Female rostrum with sparse small punctures, with sparse recumbent setae, only basally with few scales. Female abdominal ventrites 1-2 medially flat; ventrite 5 flat.

##### Material examined.

Male, holotype (QMBA): ARC3707 (EMBL # LN888189), Queensland, W Bloomfield, Mt. Misery, S15°52.706', E145°13.383',750-850 m, 30-IV-2014. Paratypes (QMBA, SMNK): Queensland: 5 exx, ARC3706 (EMBL # LN888188), ARC3708 (EMBL # LN888190), ARC3709 (EMBL # LN888191), same data as holotype; 1 ex, Mt. Misery, summit, 15°52', S 145°14', E, 850 m, 03-I-1991, Pyrethrum knockdown; 1 ex, Massey Ra., 6 km NW of Bellenden Kerr, 17°14', S 145°48', E, 1150 m, 11-X-1991.

##### Distribution.

Queensland: Mt. Misery, Massey Range.

##### Biology.

Beaten from foliage in relatively dry forest.

##### Etymology.

This epithet is an adjective based on the Cape York Peninsula, where the type locality is located.

##### Notes.

*Trigonopterus yorkensis* Riedel, sp. n. was coded as “*Trigonopterus* sp. 552”.

## Discussion

The most recent description of an Australian *Trigonopterus* species prior to this study was by Lea in 1928, reflecting a general taxonomic neglect of Australian Cryptorhynchinae, and in particular of the small sized *Trigonopterus*. The hitherto described species are found on foliage, whereas all the edaphic species dwelling in the leaf-litter are undescribed – a result agreeing with observations on other groups of tropical insects (Stork et al. 2008). Many of the new edaphic species are endemic to small areas of tropical forest on mountains of the Cape York Peninsula. Most likely, wingless weevils are sensitive to environmental changes, e.g. a warming climate (Staunton et al. 2014), and, considering their high level of endemism, they should be of concern to conservation.

The Australian *Trigonopterus* fauna is divided into a few species-groups, each restricted to geographical areas and specific life-styles: the edaphic fauna inhabiting leaf litter is shared among the *Trigonopterus australis* and *Trigonopterus bisinuatus*-groups. The former ranges with three species from Cooktown to the Iron Range, whereas the 16 species of the latter occur between Mission Beach and the Mt. Finnigan area. A few less diverse species-groups found on foliage are restricted to the northern Cape York, i.e. the *Trigonopterus nasutus*-group (a single species from the Iron Range) and the *Trigonopterus illitus*-group (three species in the area north of Cooktown). The *Trigonopterus politus* and the *Trigonopterus squamosus*-groups are relatively widespread and can be found on foliage in coastal areas ranging from northern New South Wales to the Cape York Peninsula. The taxonomy of these two species groups is problematic and could not be dealt with adequately herein, because male genital as well as external characters are relatively uniform among different species. This situation is unfortunate as the *Trigonopterus politus*-group comprises the greatest ecological band width of the Australian *Trigonopterus* — its numerous species occur in wet rainforests as well as savannah habitats. Furthermore, the *Trigonopterus politus*-group represents the largest portion of Australian *Trigonopterus* specimens stored in collections; in many cases these are incorrectly identified, if identified at all. Therefore, identification records of *Trigonopterus aequalis* Pascoe, *Trigonopterus albidosparsus* Lea and *Trigonopterus evanidus* Pascoe should be treated with caution. Presumably a dense sampling of specimens with molecular data covering the east coast of Queensland and northern New South Wales would be the most efficient way to delineate species boundaries reliably. Thus, a solution of these taxonomic problems mainly depends on freshly collected material suitable for DNA sequencing. The geographical ranges and ecologies of these “difficult species” will become sufficiently clear with such a study, hopefully allowing the safe identification of all the unnamed specimens stored in museum collections.

### Preliminary key to the *Trigonopterus* species of Australia

**Table:** 

1	Species found on foliage; elytral striae distinct or indistinct, but never deeply incised	**2**
–	Species found in the litter layer; elytral striae deeply incised	**14**
2(1)	Eyes in lateral position; forehead as wide as, or wider than rostrum. (*Trigonopterus politus*-gr.)	**9**
–	Eyes in dorsolateral position; forehead narrower than rostrum	**3**
3(2)	Elytra ferruginous, densely squamose unless partly abraded	**4**
–	Elytra black, nude (or almost nude with sparse inconspicuous scales inserted in punctures in *Trigonopterus yorkensis* Riedel, sp. n.)	**5**
4(3)	Penis parallel-sided, rounded at apex.	***Trigonopterus squamosus* Lea**
–	Penis with sides weakly diverging from base to rounded apex	***Trigonopterus striatipennis* Lea**
5(3)	Elytra subglabrous except for row of punctures at base and humeri, never with scales. Tarsi black	**6**
–	Pronotum and elytra punctate; punctures each with one narrow brown recumbent scale. Tarsi ferruginous	***Trigonopterus yorkensis* Riedel, sp. n.**
6(5)	Prothorax dorsolaterally with distinct edge; near procoxa with patch of white scales. Body size large, 3.22–3.81 mm	***Trigonopterus rostralis* Lea**
–	Prothorax evenly rounded towards sides, near procoxa without patch of white scales. Body size smaller, 1.96–3.47 mm	**7**
7(6)	Pronotum dorsally with coarse punctures (Fig. 19a)	***Trigonopterus laetus* (Lea)**
–	Pronotum dorsally with minute punctures (Figs 4a, 25a)	**8**
8(7)	Body size larger, 2.98–3.47 mm. Rostrum in male basally markedly swollen. Prothorax in front of procoxa with acute process	***Trigonopterus australinasutus* Riedel, sp. n.**
–	Body size smaller, 2.16–2.68 mm. Rostrum in male basally simple. Prothorax in front of procoxa simple	***Trigonopterus allaetus* Riedel, sp. n.**
9(2)	Rostrum with sharp median and pair of submedian ridges. Profemur dentate	***Trigonopterus sculptirostris* (Lea)**
–	Rostrum dorsally flat or with low costae, never carinate. Profemur edentate	**10**
10(9)	Elytra ferruginous. In montane habitats of Mt. Spurgeon and Mt. Finnigan	***Trigonopterus finniganensis* Riedel, sp. n.**
–	Elytra black. Usually in lowland habitats	**11**
11 (10)	Apex of mesotibia in male with two separate teeth, i.e. outer uncus and inner premucro	**12**
–	Apex of mesotibia in male with only one relatively wide tooth, apically simple or bifid	**13**
12(11)	Elytra with fine but distinct striae. Pronotum and elytra between punctures microreticulate	***Trigonopterus aequalis* Pascoe**
–	Elytra with striae invisible from most directions. Pronotum and elytra between punctures not microreticulate	***Trigonopterus evanidus* Pascoe**
13(11)	Pronotum uniformly densely punctate with relatively large punctures, dorsally and laterally of almost equal size. Apex of mesotibia in male with apically bifid tooth	***Trigonopterus cooktownensis* Riedel, sp. n.**
–	Pronotum dorsally with minute punctures, laterally with large punctures. Apex of mesotibia in male with one apically simple tooth.	***Trigonopterus albidosparsus* Lea**
14(1)	Elytral intervals irregularly costate-carinate; sutural interval basally swollen; rostrum in apical third with median denticle	***Trigonopterus australis* Riedel, sp. n.**
–	Elytral intervals costate or flat; sutural interval basally simple. Rostrum at most along apical margin with denticles, but simple further behind	**15**
15(14)	Base of rostrum in profile with distinct angulation	**19**
–	Base of rostrum in profile without distinct angulation; with shallow constriction or evenly convex to forehead	**16**
16(15)	Pronotum without or with indistinct subapical constriction. Elytral intervals costate without forming sharp prominent ridges	**17**
–	Pronotum with distinct subapical constriction. Elytral intervals costate-carinate, forming sharp and/or prominent ridges	**18**
17(16)	Body black with elytra cuneiform and pronotum subquadrate (Fig. 11a), Length ca. 2.98 mm	***Trigonopterus deplanatus* Riedel, sp. n.**
–	Body largely ferruginous, subovate (Fig. 23a); smaller, 1.92–2.20 mm	***Trigonopterus mossmanensis* Riedel, sp. n.**
18(16)	Elytra ferruginous. Epistome posteriorly with 4 denticles	***Trigonopterus fraterculus* Riedel, sp. n.**
–	Elytra black. Epistome posteriorly with curved ridge	***Trigonopterus terraereginae* Riedel, sp. n.**
19(15)	Pronotum subtrapezoidal, with sides markedly converging from base to apex; disc densely foveate-reticulate, without median costa	**20**
–	Pronotum with sides subparallel or weakly converging to preapical constriction; disk always with median costa	**21**
20(19)	Elytral intervals flat to weakly costate. Penis (Fig. 22b) with shorter, spiniform transfer apparatus	***Trigonopterus monteithi* Riedel, sp. n.**
–	Elytral intervals costate. Penis (Fig. 16b) with longer, flagelliform transfer apparatus	***Trigonopterus hasenpuschi* Riedel, sp. n.**
21(19)	Body small, pronotum plus elytron 1.90–2.40 mm, relatively compact; elytral striae weakly incised or marked by rows of isolated punctures, intervals weakly costate	**22**
–	Body larger, pronotum plus elytron 2.42–3.28 mm, more elongate; elytral striae deeply incised, intervals distinctly costate or carinate	**23**
22(21)	Elytral striae anteriorly marked by rows of large punctures. Pronotum with interspaces between punctures polished	***Trigonopterus hartleyensis* Riedel, sp. n.**
–	Elytral striae weakly incised, without large punctures. Pronotum with interspaces between punctures dull, with silky luster	***Trigonopterus boolbunensis* Riedel, sp. n.**
23(21)	Metaventrite and abdominal ventrite 1 with dense erect setae, abdominal ventrite 2 with erect scales. Ductus ejaculatorius near insertion to transfer apparatus sclerotized, forming S-shaped ribbon longer than flagellum	***Trigonopterus oberprieleri* Riedel, sp. n.**
–	Metaventrite and abdominal ventrite 1 at most sparsely setose with recumbent setae. Ductus ejaculatorius near insertion to transfer apparatus membranous; if sclerotized, slender, not ribbon-shaped	**24**
24(23)	Pronotum with pair of patches of sparse yellow scales. Elytra basally almost nude, in apical half with scattered scales. Penis subapically with lateral subangular extensions (Figs 6b, 15b, 18b)	**30**
–	Pronotum and elytra without patches of yellow scales; if sparse patches of scales present, color of scales white. Penis in apical 1/3 without lateral extensions (e.g., Figs 7b, 10b, 20b)	**32**
30(29)	Body (Fig. 15a) broader, with marked constriction between pronotum and elytra. Penis with flagellum shorter (Fig. 15b)	***Trigonopterus garradungensis* Riedel, sp. n.**
–	Body (Figs 6a, 18a) more slender, with shallow constriction between pronotum and elytra. Penis with flagellum longer (Figs 6b, 18b)	**31**
31(30)	Metatibia in apical third with blunt suprauncal projection. Flagellum ca. 3.0× longer than body of penis (Fig. 18b)	***Trigonopterus kurandensis* Riedel, sp. n.**
–	Metatibia in apical third simple, without suprauncal projection. Flagellum ca. 1.5× longer than body of penis (Fig. 6b). Daintree N.P. and Windsor Tableland	***Trigonopterus bisignatus* Riedel, sp. n.**
32(24)	Elytra cuneiform, from broad humeri converging to narrow apex	**33**
–	Elytra subovate or subparallel	**34**
33(32)	Body more slender (Fig. 7a). Pronotum coarsely sculptured, submedially interspaces confluent forming irregular wrinkles besides median ridge. From Atherton Tablelands southwards to Wooroonooran N.P.	***Trigonopterus bisinuatus* Riedel, sp. n.**
–	Body broader (Fig. 10a). Pronotum densely punctate-reticulate, with simple median costa. Daintree N.P.	***Trigonopterus daintreensis* Riedel, sp. n.**
34(32)	Body subovate (Figs 20a, 26a), with sparse vestiture dominated by narrow ochre-colored scales. Elytral intervals costate-carinate throughout	**35**
–	Body parallel-sided (Figs 3a, 21a), with sparse vestiture dominated by white scales. Elytral intervals in basal half partly transversely confluent	**36**
35(34)	Penis (Fig. 20b) with large X-shaped sclerite and complex transfer apparatus	***Trigonopterus lewisensis* Riedel, sp. n.**
–	Penis (Fig. 26b) with flagelliform transfer apparatus	***Trigonopterus robertsi* Riedel, sp. n.**
36(34)	Elytral striae with coarse punctures; intervals weakly carinate. Penis (Fig. 21b) with widened, subangulate apex; transfer apparatus with pair of triangular sclerites	***Trigonopterus montanus* Riedel, sp. n.**
–	Elytral striae with punctures less distinct; intervals costate. Penis (Fig. 3b) with subtruncate apex; transfer apparatus subrotund	***Trigonopterus athertonensis* Riedel, sp. n.**

### Catalogue of species groups of *Trigonopterus* Fauvel in Australia

***Trigonopterus australis*-group**: *Trigonopterus australis* sp. n., *Trigonopterus fraterculus* sp. n., *Trigonopterus terraereginae* sp. n.

***Trigonopterus politus*-group**: *Trigonopterus aequalis* (Pascoe), *Trigonopterus albidosparsus* (Lea), *Trigonopterus cooktownensis* sp. n., *Trigonopterus evanidus* (Pascoe), *Trigonopterus finniganensis* sp. n., *Trigonopterus sculptirostris* (Lea)

*Trigonopterus nasutus*-group*: *Trigonopterus australinasutus* sp. n.

***Trigonopterus squamosus*-group**: *Trigonopterus squamosus* (Lea), *Trigonopterus striatipennis* (Lea), *Trigonopterus yorkensis* sp. n.

*Trigonopterus illitus*-group*: *Trigonopterus allaetus* sp. n., *Trigonopterus laetus* (Lea), *Trigonopterus rostralis* (Lea)

***Trigonopterus bisinuatus*-group**: *Trigonopterus athertonensis* sp. n., *Trigonopterus bisignatus* sp. n., *Trigonopterus bisinuatus* sp. n., *Trigonopterus boolbunensis* sp. n., *Trigonopterus daintreensis* sp. n., *Trigonopterus deplanatus* sp. n., *Trigonopterus garradungensis* sp. n., *Trigonopterus hasenpuschi* sp. n., *Trigonopterus hartleyensis* sp. n., *Trigonopterus kurandensis* sp. n., *Trigonopterus lewisensis* sp. n., *Trigonopterus montanus* sp. n., *Trigonopterus monteithi* sp. n., *Trigonopterus mossmanensis* sp. n., *Trigonopterus oberprieleri* sp. n., *Trigonopterus robertsi* sp. n.

*note: the *Trigonopterus illitus*-group was not distinguished from the *Trigonopterus nasutus*-group by Riedel et al (2013b); however, based on recent analysis of molecular data, it represents a separate lineage.

## Supplementary Material

XML Treatment for Trigonopterus

XML Treatment for Trigonopterus aequalis

XML Treatment for Trigonopterus albidosparsus

XML Treatment for Trigonopterus allaetus

XML Treatment for Trigonopterus athertonensis

XML Treatment for Trigonopterus australinasutus

XML Treatment for Trigonopterus australis

XML Treatment for Trigonopterus bisignatus

XML Treatment for Trigonopterus bisinuatus

XML Treatment for Trigonopterus boolbunensis

XML Treatment for Trigonopterus cooktownensis

XML Treatment for Trigonopterus daintreensis

XML Treatment for Trigonopterus deplanatus

XML Treatment for Trigonopterus evanidus

XML Treatment for Trigonopterus finniganensis

XML Treatment for Trigonopterus fraterculus

XML Treatment for Trigonopterus garradungensis

XML Treatment for Trigonopterus hasenpuschi

XML Treatment for Trigonopterus hartleyensis

XML Treatment for Trigonopterus kurandensis

XML Treatment for Trigonopterus laetus

XML Treatment for Trigonopterus lewisensis

XML Treatment for Trigonopterus montanus

XML Treatment for Trigonopterus monteithi

XML Treatment for Trigonopterus mossmanensis

XML Treatment for Trigonopterus oberprieleri

XML Treatment for Trigonopterus robertsi

XML Treatment for Trigonopterus rostralis

XML Treatment for Trigonopterus sculptirostris

XML Treatment for Trigonopterus squamosus

XML Treatment for Trigonopterus striatipennis

XML Treatment for Trigonopterus terraereginae

XML Treatment for Trigonopterus yorkensis

## References

[B1] Alonso-Zarazaga MA Lyal CHC (1999) A world catalogue of families and genera of Curculionoidea (Insecta: Coleoptera) (excepting Scolytidae and Platypodidae). Entomopraxis, Barcelona, 315 pp.

[B2] Beutel RG Leschen RAB (2005) Handbook of Zoology (Vol. IV, Part 38, Coleoptera, Beetles) – Vol. 1: Morphology and Systematics (Archostemata, Adephaga, Myxophaga, Polyphaga partim). Walter de Gruyter, Berlin, 567 pp.

[B3] Besuchet C Burckhardt DH Löbl I (1987) The “Winkler/Moczarski” eclector as an efficient extractor for fungus and litter coleoptera. The Coleopterists Bulletin 41: 392–394.

[B4] Fauvel A (1862) Coléoptères de la Nouvelle-Calédonie, recueillis par M. E. Déplanche, chirurgien de la marine impériale (1858-59-60). Bulletin de la Société Linnéenne de Normandie 7: 120–185.

[B5] Lea AM (1913) Revision of the Australian Curculionidae belonging to the subfamily Cryptorhynchinae. Part XI. Proceedings of the Linnean Society of New South Wales 37: 602–616.

[B6] Lea AM (1928) Australian Curculionidae of the subfamilies Haplonycides and Cryptorhynchides. Transactions and Proceedings of the Royal Society of South Australia 52: 95–164.

[B7] Leschen RAB Beutel RG Lawrence JF Slipinski A (2009) Handbook of Zoology (Vol. IV, Part 38, Coleoptera, Beetles) – Vol. 2: Morphology and Systematics (Elateroidea, Bostrichiformia, Cucujiformia partim). Walter de Gruyter, Berlin, 786 pp.

[B8] Pascoe FP (1872) Additions to the Australian Curculionidae. Part III. Annals and Magazine of Natural History (Series 4) 10: 84–101.

[B9] Pullen KR Jennings D Oberprieler RG (2014) Annotated catalogue of Australian weevils (Coleoptera: Curculionoidea). Zootaxa 3896: 1–481. doi: 10.11646/zootaxa.3896.1.1 25543671 10.11646/zootaxa.3896.1.1

[B10] Riedel A (2005) Digital imaging of beetles (Coleoptera) and other three-dimensional insects. In: Häuser C Steiner A Holstein J Scoble MJ (Eds) Digital Imaging of Biological Type Specimens. A Manual of Best Practice. Stuttgart Results from a study of the European Network for Biodiversity Information, 222–250.

[B11] Riedel A (2010) One of a thousand - a new species of *Trigonopterus* (Coleoptera, Curculionidae, Cryptorhynchinae) from New Guinea. Zootaxa 2403: 59–68. doi: 10.1111/j.1463-6409.2009.00404.x

[B12] Riedel A Daawia D Balke M (2010) Deep cox1 divergence and hyperdiversity of *Trigonopterus* weevils in a New Guinea mountain range (Coleoptera, Curculionidae). Zoologica Scripta, 39(1): 63–74.

[B13] Riedel A Sagata K Suhardjono YR Tänzler R Balke M (2013a) Integrative taxonomy on the fast track - towards more sustainability in biodiversity research. Frontiers in Zoology 10: . doi: 10.1186/1742-9994-10-15 10.1186/1742-9994-10-15 PMC3626550 23537182

[B14] Riedel A Sagata K Surbakti S Tänzler R Balke M (2013b) One hundred and one new species of *Trigonopterus* weevils from New Guinea. ZooKeys 280: 1–150. doi: 10.3897/zookeys.280.3906 23794832 10.3897/zookeys.280.3906 PMC3677382

[B15] Riedel A Tänzler R Balke M Rahmadi C Suhardjono YR (2014) Ninety-eight new species of *Trigonopterus* weevils from Sundaland and the Lesser Sunda Islands. ZooKeys 467: 1–162. doi: 10.3897/zookeys.467.8206 25610340 10.3897/zookeys.467.8206 PMC4296478

[B16] Staunton KM Robson SK Burwell CJ Reside AE Williams SE (2014) Projected distributions and diversity of flightless ground beetles within the Australian Wet Tropics and their environmental correlates. PLoS ONE 9(2): . doi: 10.1371/journal.pone.0088635 10.1371/journal.pone.0088635 PMC3930578 24586362

[B17] Stork NE Grimbacher PS Storey R Oberprieler RG Reid C Slipinski S (2008) What determines whether a species of insect is described? Evidence from a study of tropical forest beetles. Insect Conservation and Diversity 1(2): 114–119. doi: 10.1111/j.1752-4598.2008.00016.x

[B18] Tänzler R Sagata K Surbakti S Balke M Riedel A (2012) DNA barcoding for community ecology - how to tackle a hyperdiverse, mostly undescribed Melanesian fauna. PLoS ONE 7(1): . doi: 10.1371/journal.pone.0028832 10.1371/journal.pone.0028832 PMC3258243 22253699

[B19] Tänzler R Toussaint EFA Suhardjono YR Balke M Riedel A (2014) Multiple transgressions of Wallace’s Line explain diversity of flightless *Trigonopterus* weevils on Bali. Proceedings of the Royal Society B: Biological Sciences 281: . doi: 10.1098/rspb.2013.2528 10.1098/rspb.2013.2528 PMC3973253 24648218

[B20] Tänzler R van Dam MH Toussaint EFA Suhardjono YR Balke M Riedel A (2016) Macroevolution of hyperdiverse flightless beetles reflects the complex geological history of the Sunda Arc. Scientific Reports 5: . doi: 10.1038/srep18793 10.1038/srep18793 PMC4732383 26742575

[B21] van de Kamp T Vagovič P Baumbach T Riedel A (2011) A biological screw in a beetle´s leg. Science 333(6038): 52. doi: 10.1126/science.1204245 10.1126/science.1204245 21719669

[B22] van de Kamp T dos Santos Rolo T Vagovič P Baumbach T Riedel A (2014) Three-dimensional reconstructions come to life – interactive 3D PDF animations in functional morphology. PLoS ONE 9(7): . doi: 10.1371/journal.pone.0102355 10.1371/journal.pone.0102355 PMC4100761 25029366

[B23] van de Kamp T Cecilia A dos Santos Rolo T Vagovič P Baumbach T Riedel A (2015) Comparative thorax morphology of death-feigning flightless cryptorhynchine weevils (Coleoptera: Curculionidae) based on 3D reconstructions. Arthropod Structure & Development 44: 509–523. doi: 10.1016/j.asd.2015.07.004 26259678 10.1016/j.asd.2015.07.004

[B24] Zimmerman EC (1992) Australian Weevils (Coleoptera: Curculionoidea). CSIRO Australia, Melbourne, 707 pp [Vol. VI. Colour Plates 305–632]

